# Multiple Indicators of Undernutrition, Infection, and Inflammation in Lactating Women Are Associated with Maternal Iron Status and Infant Anthropometry in Panama: The MINDI Cohort

**DOI:** 10.3390/nu14173497

**Published:** 2022-08-25

**Authors:** Doris González-Fernández, Elizabeta Nemeth, Emérita del Carmen Pons, Odalis Teresa Sinisterra, Delfina Rueda, Lisa Starr, Veena Sangkhae, Enrique Murillo, Marilyn E. Scott, Kristine G. Koski

**Affiliations:** 1 School of Human Nutrition, McGill University, (Macdonald Campus), Ste-Anne de Bellevue, QC H9X 3V9, Canada; 2 Center for Iron Disorders, David Geffen School of Medicine, University of California, Los Angeles, CA 90089, USA; 3 Department of Nutritional Health, Panamanian Ministry of Health, Panama City, Panama; 4 “Panamá Norte” Health Region, Panamanian Ministry of Health, Panama City, Panama; 5 “Comarca Ngäbe-Buglé” Health Region, Panamanian Ministry of Health, Panama City, Panama; 6 Department of Biochemistry, University of Panama, Panama City, Panama; 7 Institute of Parasitology, McGill University, (Macdonald Campus), Ste-Anne de Bellevue, QC H9X 3V9, Canada

**Keywords:** anemia, lactating women, serum iron, ferritin, serum transferrin receptor, inflammation, hepcidin, supplementation, undernutrition, infant anthropometry

## Abstract

Maternal infections, nutrient deficiencies, and inflammation (MINDI) co-exist in lactating indigenous women in Panama, but their impact on maternal iron status and infant growth is unknown. For this secondary analysis of cross-sectional data of lactating mothers from our MINDI cohort, we investigated associations of MINDI variables with maternal anemia, elevated serum transferrin receptor (sTfR), low serum iron, hepcidin, ferritin, and infant weight-for-age (WAZ), length-for-age (LAZ), and head-circumference-for-age (HCAZ) Z-scores in 99 mother-infant dyads. A bootstrapping resampling procedure preselected covariates for inclusion in multivariable regressions models from chronic maternal infections and nutritional status [folate, vitamins A, D, retinol-binding protein (RBP), insulin-growth factor-1 (IGF-1)] and inflammation [C-reactive protein (CRP), cytokines, platelet indices] indicators. Anemia was prevalent (53.5%) but underestimated due to widespread low plasma volume (<2.2 L, 79.9%) and was associated with indicators of malnutrition [lower IGF-1, body mass index (BMI), vitamin D, and intake of green/leafy vegetables], but not inflammation. Higher CRP was associated with lower serum iron, and higher hepcidin and ferritin, whereas maternal platelets were associated with lower HCAZ (β = −0.22), WAZ (β = −0.17), and LAZ (β = −0.17). Higher LAZ was also associated with maternal serum vitamin D (β = 0.23), whereas maternal iron supplementation lowered LAZ (β = −0.22). Assessment of iron status in this MINDI cohort is complex and supplementation strategies must consider consequences for both the mother and the infant.

## 1. Introduction

Anemia is a major problem in lactation as blood loss at delivery is considered one of the main causes of anemia [1]. Also, due to higher nutritional requirements, lactating women are at increased risk of multiple nutrient deficiencies [2] including folic acid and vitamin B_12_ [3], vitamin A [4], and D [5]. Anemia of malnutrition has also been associated with both protein and calorie malnutrition [6,7]. In lactating women in developing countries, chronic infection-related inflammation [8] contributes to anemia [7,9]. Anemia of chronic inflammation manifests as low serum iron with normal iron stores [10]. It is the consequence of iron restriction due to inflammation that is mediated by the hepatic release of hepcidin, which blocks intestinal iron absorption and favors retention of iron in macrophages [11]. This increase in serum hepcidin has been used to distinguish anemia of iron deficiency from anemia of inflammation in children [12] and during pregnancy [13], but we are aware of only one other study from Ethiopia on the impact of inflammation on anemia in lactating women [14].

Currently, the World Health Organization (WHO) recommends the use of both serum ferritin and hemoglobin (Hb) [15] to evaluate iron status in population studies. However, because ferritin is increased by inflammation [16], approaches such as increasing the cutoff that defines iron deficiency, excluding from analysis individuals with an elevated acute phase indicator, usually C-Reactive Protein (CRP) [15,17], or using a regression for adjusting ferritin for the presence of infection/inflammation [18] have been recommended. Serum iron has also been used as an iron status indicator in population studies in lactating women [19], but serum iron concentrations are known to decrease with inflammation [15,20]. Elevated soluble transferrin receptor (sTfR) diagnostic of iron deficiency and linked with erythropoiesis has also been used in population studies on the assumption that sTfR is not influenced by inflammation [15]. However, elevations in sTfR can signal increased erythropoiesis resulting from either hemolysis or ineffective erythropoiesis [21]. Moreover, the assumption that sTfR is not influenced by inflammation has been challenged and an equation for adjusting sTfR for inflammation and infection has been proposed [22]. 

In Panama, anemia is a significant public health problem with a prevalence of 40% [23]. Our previous studies in lactating women in this remote, indigenous Ngäbe-Buglé region reported multiple infections [24] and deficiencies of vitamin A (18.5%), folic acid (31.3%), vitamins B_12_ (46.4%), and D (68.7%) [25], despite government supplementation programs that provide folic acid (400 µg/d) during pregnancy, iron (60 mg/d) during pregnancy and lactation, vitamin A (200,000 IU) after delivery, and a multiple-nutrient supplement (MNS) to pregnant and lactating women who are not overweight [26]. We also observed that even though 97% of women had at least two co-occurring infections, CRP was only elevated in 30% using a cut-off of >3 mg/L, and that the complex set of interacting infections and nutrient deficiencies modulated the concentration of CRP. For example, folic acid deficiency and severity of vaginal trichomoniasis were associated higher CRP, whereas eosinophilia, characteristic of intestinal nematode infections, was associated with lower CRP concentrations [25]. Furthermore, despite nearly universal iron supplementation, anemia persisted in pregnant [27] and women of reproductive age [28], experiencing a range of co-existing infections and multiple nutrient deficiencies [25]. 

There is also evidence that both maternal malnutrition and inflammation can influence infant growth in early lactation. Although breastmilk is recommended as the sole source of nutrition for infants ≤ 6 months, milk constituents can vary depending on maternal nutritional status and affect infant growth [29]. Children of underweight mothers from Ethiopia were 1.20 times more likely to be stunted and 1.52 times more likely to be wasted, and those from mothers with anemia were 1.18 times more likely to be stunted [30]. Moreover, concentrations of breastmilk micronutrients including sodium, calcium, magnesium, and potassium were positively associated with infant anthropometry in a Guatemalan study [31]. On the other hand, breastmilk cytokines IL-6 and TNF-α have been negatively associated with infant weight-for-age at 2–3 months of age [32], and the presence of subclinical mastitis, often associated with inflammation [33] increased the odds of underweight and stunting in exclusively breastfed Guatemalan infants at 6 weeks of age [34]. Moreover, there is emerging evidence that maternal iron supplementation may negatively affect growth [35].

Given that lactating women from these Panamanian communities had multiple infections, nutrient deficiencies, and inflammation (MINDI) [24,25], we suspected a multi-factorial origin of anemia. Therefore, our objectives were to (1) identify determinants of anemia and other iron status indicators in lactating women with co-occurring chronic infections (oral, skin, intestinal, and vaginal) and inflammation, and with co-existing multiple nutrient deficiencies; (2) assess the ranked dominance of foods, nutritional deficiencies, inflammation markers, and duration and type of supplementation on iron status indicators; and (3) to determine if indicators of maternal iron status, inflammation/infection, or maternal supplementation were associated with infant growth during the first 6 months of lactation. A secondary objective was to also explore if the duration of iron supplementation benefited the mother or might have consequences for the infant given growing concerns about its limited efficacy and possible harm in developing countries [29].

## 2. Materials and Methods

### 2.1. Study Design and Participants

This cross-sectional study conducted between August and November 2010 recruited 99 indigenous lactating women during the first 6 months of lactation. Our target population was all lactating mother–infant dyads, who attended their monthly ambulatory postpartum follow-up visit at the local health center, often requiring a 1–2 h walk, as previously described [24]. Briefly, all women belonging to the Ngäbe-Buglé Health Region who had a singleton delivery in the past 6 months were invited to participate and through collaboration from the nurses/midwives, we were able to recruit most if not all targeted women from several remote communities. Most lived in extreme poverty and were food insecure [36]. Ethical approval was obtained from The Gorgas Memorial Institute in Panama and from McGill University. At recruitment, data on obstetric history (number of gestations, parity, date of last menses, and date and place of delivery) were obtained from maternal interviews and from their pregnancy follow-up card. At the time of visit, women were asked how many times in the past week (portions/week) they had eaten the following three food groups: animal-source foods, green/leafy vegetables, and orange/red fruits or vegetables. We also asked if women were taking supplements (iron and/or MNS). If they reported taking iron, we obtained information on dosage and duration from mothers’ recall and confirmed from the pregnancy follow-up card, completed by local Ministry of Health nurses. Women receiving MNS provided information on the amount of product (tbsp/d) used for consumption. The clinical presence of caries and skin infections, and presence and severity of urogenital infections (urine leukocyte esterase and vaginal smears) were evaluated by a physician. At the same visit, samples for laboratory analyses and maternal–infant anthropometry were also taken.

### 2.2. Laboratory Measures

All biologic samples were taken the day of the interview. Laboratory measurements were processed at several locations: San Felix Hospital in rural Panama; the Gorgas Institute and INDICASAT in Panama City; McGill University; and most recently, the Center for Iron Disorders of UCLA. We measured complete blood cell count (BC-5500 Mindray Auto Hematology Analyzer, Huntingdon, UK) at San Felix hospital, and serum concentrations of ferritin (ELISA, MP Biomedicals, Sydney, Australia) and sTfR (RAMCO immunoassay), from previously frozen serum, were processed at the Gorgas Institute, and vitamin A (HPLC) at INDICASAT in Panama City within the 3 months following blood collection. At McGill University, concentrations of 25 OH-vitamin D3 (LIAISON, DiaSorin, Vercelli, Italy), folic acid and vitamin B_12_ (chemiluminescence, MODULAR E170, Roche Diagnostics, Basel, Switzerland), retinol-binding protein (RBP, ELISA, MP Biomedicals), and insulin-growth factor-1 (IGF-1, Human IGF-1 single plex, Millipore Corporation Canada, Toronto, Canada) were measured and used as indicators of nutritional status [37]. Also at McGill, several indicators of inflammation were measured, including CRP (ELISA, MP Biomedicals), interleukin (IL)1β, IL4, IL6, IL10, IL12, IL13, IL17, interferon (INF)ϒ, tumor necrosis factor (TNF)α, and monocyte chemoattractant protein 1 (MCP-1) (Luminex Magnetic Bead Panel, Millipore, Burlington, MA, USA). Inflammation was defined as CRP > 5 mg/L [38], and eosinophilia as eosinophils > 600 × 10^3^/mm^3^ [39]. Nutritional and inflammation biomarkers were analyzed within 6 months after serum collection. More recently, hepcidin (Intrinsic Hepcidin IDx™ ELISA Kit, Intrinsic LifeSciences, CA, USA) and serum iron (spectrophotometry, FERENE^®^-ENDPOINT) were processed at the UCLA Center for Iron Disorders from the bio-banked serum samples in 2019.

To account for changes in hemoglobin with different post-partum stages, we used the cut-off for anemia during pregnancy (<110 g/L) in the first 8 weeks post-partum, as suggested by Milman [1]. For women > 8 weeks post-partum, the cut-off was set as <120 g/L as is the case for non-pregnant women [15]. To assess nutritional deficiencies, we defined low hematocrit (<36%) [40], folic acid (<10 nmol/L) [41], vitamin B_12_ (<150 pmol/L) [41],vitamin D (<50 nmol/L) [42], and vitamin A (retinol < 1.05 µmol/L) [43]. Protein deficiency was defined as RBP < 30 mg/L [37], and low iron status if either serum iron < 8.9 µmol/L [15], or sTfR > 8.3 mg/L (laboratory kit RAMCO^®^, Chennai, India), or ferritin < 70 µg/L (as for populations with infection/inflammation) [38] were present. We also report ferritin < 15 µg/L (suggested for women without inflammation) and the cut-off < 30 µg/L used by the Panamanian Ministry of Health for adjusting for inflammation [44].

As an indicator of vitamin A deficiency post-partum, the retinol/RBP molar ratio may be used to measure retinol saturation [45,46] and at the same time can be used to assess the effectiveness of vitamin A supplementation. In this case, a retinol/RBP molar ratio (usually 1:1 [46]) is an indicator of retinol saturation, whereas a ratio < 0.8 mol/mol is considered low saturation [47]. On the other hand a ratio greater than 1 may signal high concentrations of unbound vitamin that may occur with megadose vitamin A supplementation and might theoretically be exacerbated if RBP is low [48,49].

### 2.3. Maternal/Infant Anthropometry

Ministry of Health nurses measured weight and height of mothers, and, at the same visit, infants’ weight (SECA balances), length (wood-stadiometers), and head circumference using unextendible measuring tapes according to Panamanian MOH guidelines [50]. Maternal body mass index (BMI, kg/m^2^) was classified as underweight (<18.5), normal (18.5–24.9), pre-obesity (25.0–29.9), or obese (class 1 between 30.0 and 34.9, class II if 35.0–39.9 or class III if ≥ 40) according to WHO standards [51]. Maternal plasma volume was calculated as total blood volume (TBV) × (1 − hematocrit) [52], where TBV was calculated using Nadler’s equation [TBV = 0.3561 × (height in meters)^3^ + 0.03308 × (weight in kg) + 0.1833] [53]. Low plasma volume was defined as < 2.2 L based on a cohort of 70 normal lactating women from the Netherlands [54]. Z-scores of infant weight-for-age (WAZ), length-for-age (LAZ), weight-for-length (WLZ), and head-circumference-for-age (HCAZ) were calculated using the STATA16 least mean squares method applied to WHO reference data [55].

### 2.4. Statistical Analyses

STATA16^®^ (StataCorp, College Station, TX, USA) was used. Maternal and infant characteristics were compared between women with and without anemia using Student’s *t*-test, Kruskal–Wallis, χ^2^, or Fisher’s exact test, depending on the nature of the variable. χ^2^ or Fisher exact tests, if any cell value in contingency tables were <5, were also used to evaluate if history of preterm birth differed by maternal anemia, iron status, or intake of MNS. Descriptive distribution graphs of iron status indicators by weeks after delivery showing fractional polynomial regression curves and cut-offs indicating anemia were run. Maternal RBC indices, iron, inflammation, and nutritional indicators were compared between women with and without protein deficiency (RBP < 30 mg/L) using Student’s *t*-test or Kruskal–Wallis test for normally and not-normally distributed variables respectively, and the Spearman correlation between RBP and vitamin A was evaluated.

#### 2.4.1. Selection of Independent Variables for Regression Models Using Bootstrapping

In order to select independent variables to include in models of low Hb, low serum iron, high sTfR, concentrations of ferritin and hepcidin, and infant anthropometry Z-scores, the following process was used. For each model, we preselected meaningful covariates using an exploratory bootstrapping procedure with a backward stepwise algorithm that counted the total number of times each variable was selected after 1000 repetitions. Only variables entering ≥500 times [56] were further used. This procedure was applied to (1) maternal characteristics [age, parity, history of home delivery, exposure to wood smoke and fieldwork, intake of animal-source foods, orange/red fruits or vegetables and green/leafy vegetables/week, intake of iron (yes/no, number of iron tablets/d and weeks taking iron) and MNS (yes/no and number of tbsp/day), BMI and plasma volume]; (2) iron status indicators [Hb, ferritin, sTfR, serum iron, hepcidin, total RBC, hematocrit (%), mean corpuscular volume (MCV, fL), mean corpuscular hemoglobin (MCH, pg), mean corpuscular hemoglobin concentration (MCHC, g/L), and red cell distribution width coefficient of variation (RDW-CV, %)]; (3) serum nutritional indicators (RBP, IGF-1, folic acid, vitamins A, B_12_, and D); (4) inflammation indicators [white blood cell (WBC) counts, and platelet indices, CRP, cytokines]; (5) vaginal microorganisms (*Lactobacillus, Bacteroides/Gardnerella, Mobiluncus, Trichomonas*, yeast, *Diplococcus*), urinary leukocyte esterase (score) as an indicator of severity of urinary infection, score of severity of caries, and presence of scabies. The retinol/RBP ratio was tested separately.

#### 2.4.2. Multivariate Model Construction

Multivariate fractional polynomial (MFP) logistic regression models for anemia, low serum iron (<8.9 µmol/L), and elevated sTfR (>8.3 mg/L) were run using the variables that met the bootstrapping cut-off (*p* < 0.05). For ferritin, for which the low number (*n* = 6) of women with ferritin ≥ 70 µg/L did not allow a logistic regression approach, and for hepcidin, which lacks established cut-offs, MFP linear regression models were applied to Log *n*-transformed continuous variables and a model for log-platelets was run to assess their association with iron deficiency and inflammation. The MFP process combines backwards elimination with optimization of fit while modeling nonlinearity of continuous variables [57]. Specifically, each continuous predictor is univariately modeled using transformations into their suitable functional forms from the fractional polynomial class, adjusting for other predictors, to obtain a simplified functional form that selects the stronger predictors [58]. MFP backwards selection of variables was set at *p* < 0.25 to avoid missing variables of importance [59]. If more than seven variables were selected, the *p*-value was decreased to allow a maximum of seven independent variables/model for compliance with a power of 0.80 and a medium effect size [60], in order to avoid overfitting.

Both bootstrapping and MFP approaches were used to run MFP linear regression models assessing associations of WAZ, LAZ, and HCAZ, adjusting for gestational age at delivery. All models were controlled for weeks after delivery. Absence of collinearity using non-significant Spearman’s correlations (Table S3) and variance inflation factor < 10, and stability of coefficients (condition number < 30) were confirmed for variables entering each model. Significance was set at *p* < 0.05. To address associations showing high leverage, we winsorized extreme observations, by replacing the highest or lowest values with the next value counting inwards from the extremes [61] for a maximum of three extreme observations (three high observations for retinol/RBP molar ratio, one high observation for plasma volume, one low observation of hematocrit). Areas under the receiving operating characteristic curve (AUC) were calculated for final MFP logistic regression models.

The relative importance of independent variables was assessed using general dominance analysis. This method assesses possible combinations of the independent variables by aggregating fit metrics across multiple models and creates an estimation model based on contribution to the statistic measuring overall fit of the model, while showing single ‘dominance’ values for each independent variable [62].

To better understand the response of ferritin, hepcidin, and serum iron to inflammation, the predicted probability of these iron indicators at different values of CRP was calculated and graphed using the ‘margins’ command in STATA, from a generalized linear model (specifying a gamma family and a log link), using the continuous iron indicator as a dependent variable and predictors from their respective MFP regression model as independent variables. Similarly, predicted probabilities of serum iron at different platelet counts were explored.

## 3. Results

### 3.1. Population Characteristics

Maternal characteristics of lactating mothers are summarized in Table 1 and Table 2. In general, women were involved in fieldwork and used wood as fuel for cooking. Women experienced a range of vaginal and urinary tract infections, caries, and scabies, and showed evidence of low-grade inflammation based on CRP. Maternal anthropometry identified no underweight women; 48.5% had normal weights, 43.4% were classified as pre-obese, and obesity class I occurred in 8.1%. Diets were low in animal-source foods, fruits, and vegetables. Women were deficient in protein, vitamin A, D, B12, folate, and iron, despite most receiving iron tablets containing 60 mg of elemental iron/d alone or together with MNS beginning during pregnancy, as well as 200,000 IU of vitamin A post-partum. Most infants (95%) were born at term and 42% were home deliveries. At enrollment, we found that 5% of mothers had a preterm delivery. All infants were exclusively or predominantly breastfed, 16.3% were stunted, 9.1% were underweight, and 5.0% had low head circumference for their age at enrollment.

**Figure 1:**
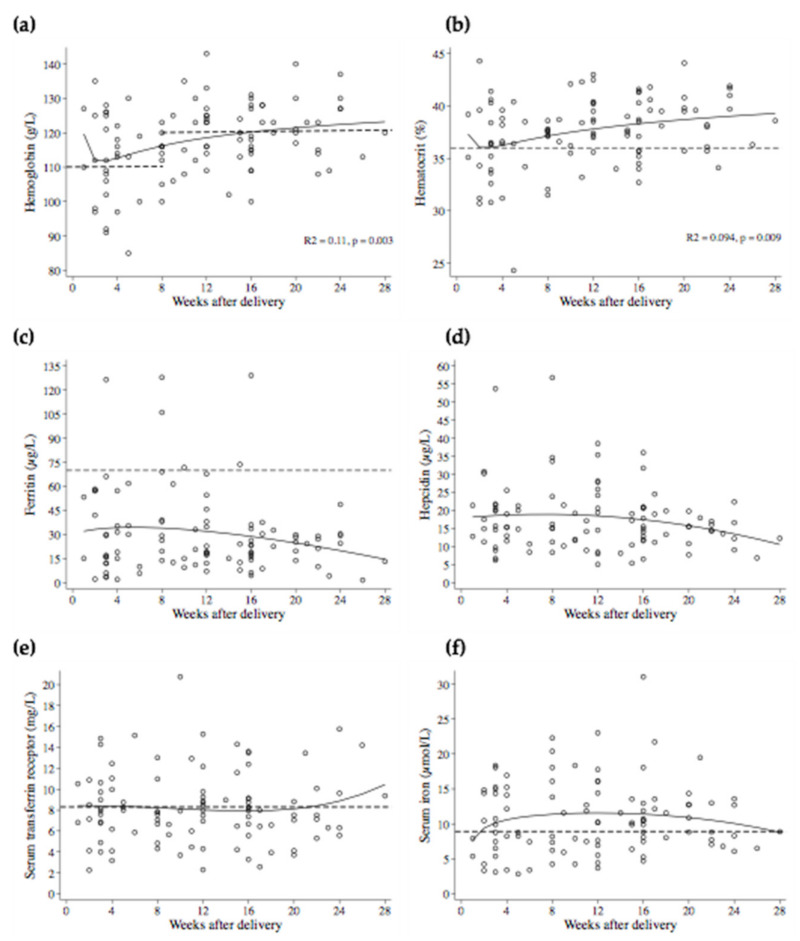
Scatter plots of iron status indicators by weeks after delivery. Solid lines show fractional polynomial regression lines. Spearman correlations showed significant correlations of (**a**) hemoglobin (r_s_ = 0.28, *p* = 0.005) and (**b**) hematocrit (r_s_ = 0.28, *p* = 0.005) with weeks post-partum, and non-significant correlations of (**c**) ferritin (r_s_ = −0.09, *p* = 0.36), (**d**) hepcidin (r_s_ = −0.12, *p* = 0.24), (**e**) serum transferrin receptor (r_s_ = −0.007, *p* = 0.94), and (**f**) serum iron (r_s_ = 0.07, *p* = 0.51) with weeks post-partum. Dashed lines indicate cut-offs for deficiencies according to WHO, except for hepcidin, for which no official cut-off is available.

The profiles of Hb, hematocrit, ferritin, hepcidin, serum iron, and sTfR with weeks post-partum are shown in Figure 1 and Table 2. Hb and hematocrit were higher as weeks post-partum increased, whereas ferritin, serum iron, sTfR, and hepcidin did not differ. Of note, 49.1% of anemic women had a hematocrit ≥ 36%, showing the presence of hemoconcentration in the population. This was further confirmed by calculating the plasma volume, which was low in most women (<2.2 L, 79.8%).

Moreover, one third of mothers (36.4%) were protein deficient (RBP < 30 mg/L) (Table 2). Protein deficient women also had lower Hb and hematocrit, as well as higher WBC, neutrophils, monocytes, and lower IL-13. No differences were found among other RBC indices, inflammation, or nutritional indicators including iron status indicators (Table S1). RBP and vitamin A concentrations were not correlated (r_s_ = 0.05, *p* = 0.63). In addition to finding that 44.4% of women had a vitamin A saturation < 0.8 indicating vitamin A deficiency, we also found that the retinol/RBP ratio was > 1 in 33%, indicating unbound vitamin A.

### 3.2. Comparison of Women with and without Anemia

Comparisons of maternal characteristics and biomarkers, and infant anthropometry between women with and without anemia, are shown in Table 3. As expected, RBC indices (RBC count, Hb, hematocrit, MCV, and MCH) were lower, and RDW higher in women with anemia. Furthermore, concentrations of serum iron, ferritin, and hepcidin were lower, and the frequency of low serum iron was higher in anemic women, whereas no difference was found in sTfR concentrations or in the frequency of elevated sTfR. Also, no differences were found between low ferritin at different cut-offs < 70, < 30 or < 15 µg/L by anemia status. Plateletcrit was higher in women with anemia, whereas none of the other indicators of inflammation (WBC, CRP, and cytokines) or infections or infant anthropometry differed between anemic and non-anemic women. Among nutritional status indicators, vitamin A concentrations were lower, and the prevalence of deficiencies in folic acid and vitamin D were higher in anemic women.

### 3.3. MFP Logistic Regression Models for Anemia, Elevated sTfR, and Low Serum Iron

Anemia: The MFP logistic regression model for anemia is shown in Table 4A. Higher values of three nutritional status indicators were associated with lower odds of anemia; these included serum iron (*p* = 0.005), vitamin A (*p* = 0.006), and IGF-1 (*p* = 0.022). In contrast, plasma volume, which ranked fourth in dominance, was the only maternal factor whose increase was associated with higher odds (OR 1.44, *p* = 0.010) of anemia, showing that hemodilution accounts for lower hemoglobin, and confirming the presence of hemoconcentration in this MINDI cohort. Of note, ferritin, hepcidin, and sTfR did not enter the model for anemia.

Elevated sTfR: The model for sTfR >8.3 mg/L is presented in Table 4B. There were no significant associations of elevated sTfR with RBC indices or other iron status indicators. Only the retinol/RBP molar ratio, which is a measure of vitamin A saturation, was associated with an increased odds of elevated sTfR (OR 2.7, *p* = 0.007). Additionally, when we entered RBP and vitamin A as separate variables in the model, only higher RBP but not vitamin A, lowered the odds of elevated sTfR, suggesting that low protein status prevailed over vitamin A as a determinant of elevated sTfR. Dominance analyses also showed that the retinol/RBP molar ratio explained half (0.499) of the overall fit statistics (0.149).

**Figure 2:**
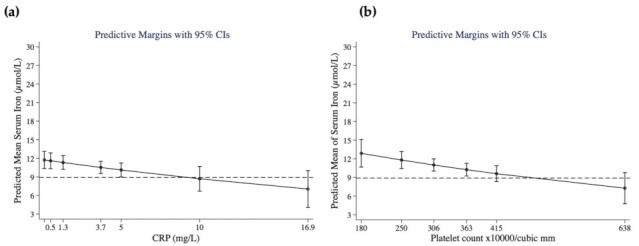
Predictive probabilities of (**a**) serum iron (µmol/L) at CRP (mg/L) quantile concentrations of 0.1 (minimum), 0.5 (25th quantile), 1.3 (median), 3.7 (75th quantile), 16.9 (maximum), and of (**b**) serum iron (µmol/L) at platelet count quantile numbers (× 10^3^/mm^3^) of 180 (minimum), 250 (25th quantile), 306 (median), 363 (75th quantile), and 638 (maximum). Additional values at CRP (5 and 10 mg/L) and platelets (415 × 10^3^/mm^3^) are shown. Predictive margins are controlled for other variables entering the MFP regression model (Table 4C). Dashed lines denote the WHO cut-off for low serum iron.

Low serum iron: The MFP logistic regression model for serum iron is shown in Table 4C. Two biomarkers of inflammation, platelets (OR 2.39, *p* = 0.012) and CRP (OR 1.29, *p* = 0.019), increased the likelihood of a serum iron concentration < 8.9 umol/L, but the expected association of higher hepcidin with increased odds of low serum iron was not observed. On the other hand, indicators of higher iron stores and nutritional status (ferritin and IGF-1) were associated with decreased odds of low serum iron. Furthermore, the predicted probability for serum iron at different quantiles of CRP and platelet counts (Figure 2) showed that at CRP = 5 mg/L, serum iron was 10.1 ± 0.6 µg/L (Figure 2A), and for platelets at 415 × 10^3^/mm^3^ (Figure 2B), serum iron was 9.60 ± 0.65 µg/L. Dominant variables in this model with an overall fit statistic of 0.357 were, in order, platelets, ferritin, CRP, and IGF-1.

### 3.4. MFP Linear Regression Model for Log Ferritin

**Figure 3:**
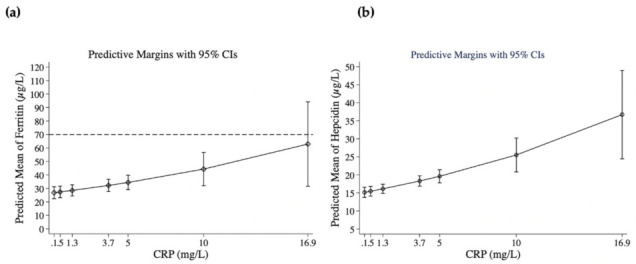
Predictive probabilities of (**a**) ferritin (µg/L) and (**b**) hepcidin (µg/L) at quantile concentrations of CRP (mg/L): 0.1 (minimum), 0.5 (25th quantile), 1.3 (median), 3.7 (75th quantile), and 16.9 (maximum). Additional values at CRP (5 and 10 mg/L) are shown. Predictive margins are controlled for other variables entering the MFP regression model for ferritin excluding hepcidin (Table 4B) and for hepcidin excluding ferritin (Table 4D). The dashed line denotes the WHO cut-off for low ferritin in populations with infection/inflammation.

The linear model for ferritin (Adj R^2^ = 0.56; overall model fit of 0.589) showed that hepcidin explained 45% of ferritin variability and nearly half of the model’s overall fit (Table 5A). Thereafter, dominance analyses showed that higher serum iron, higher hemoglobin, and lower sTfR followed in rank, and that higher CRP occupied last place, contributing only (8%) to the overall fit (Table 5A). If hepcidin was removed from the model, higher hematocrit was associated with higher ferritin, but other associations for serum iron, sTfR, and CRP were conserved (Table 5B). Predictive margins of ferritin at different CRP concentrations showed that ferritin at a concentration of 34.4 μg/L corresponded to CRP at 5 mg/L (95% CI: 29.1, 39.8) (Figure 3a).

### 3.5. MFP Linear Regression Model for Log Hepcidin

In the model for log hepcidin (Adj. R^2^ = 0.57), ferritin, which ranked first in dominance, explained 71% of the overall model fit (0.600) (Table 5C); the remaining four factors in the dominance model captured less than 25% of the model fit. To explore possible associations that could be masked by the strong hepcidin–ferritin association, a second model for hepcidin excluding ferritin (Adj. R^2^ = 0.36, overall model fit = 0.462) (Table 5D) was completed; in this case, hepcidin retained negative associations with indicators of nutritional status (intake of animal-source foods and IGF-1) and revealed positive associations with hemoglobin, serum iron, CRP, and duration of iron supplementation. For hepcidin, the predicted probability associated with a CRP of 5 mg/L corresponded to hepcidin concentration of 20 µg/L (95% CI: 17.8–21.4 µg/L) (Figure 3b). Hepcidin > 20 µg/L was present in 30.3% of women.

### 3.6. Maternal Nutritional and Inflammation Indicators but Not Iron Status Indicators were Associated with Infant Anthropometry

HCAZ: The MFP model for HCAZ (Adj R^2^ = 0.15, overall model fit = 0.201) showed that higher maternal parity was associated with larger HCAZ, whereas higher plateletcrit was associated with lower HCAZ (Table 6A). Only parity and plateletcrit, which ranked first and second in standardized dominance, were significant in the model.

LAZ: Indicators of maternal nutrition and inflammation status entered the MFP model for LAZ (Adj. R^2^ = 0.33, overall model fit = 0.379), where higher maternal plasma volume accounted for 46% of the overall model fit. Both duration of iron supplementation and higher platelet count, which ranked second and third in dominance, were associated with lower LAZ, whereas higher maternal vitamin D concentrations and intake of MNS were associated with higher LAZ, ranking fourth and fifth respectively in dominance (Table 6B).

WAZ: In the MFP model for WAZ (Adj R^2^ = 24%, overall model fit = 0.277), two maternal variables, plasma volume and higher maternal intake of animal-source foods/week, had a positive association with WAZ and together accounted for 51% of the overall fit statistics (0.277) (Table 6C). Higher platelet counts were associated with lower WAZ, ranking third in dominance after plasma volume and maternal intake of animal-source foods.

Finally, to better understand associations with LAZ and HCAZ with platelet indices, a MFP linear model for log-platelets (adj. R^2^ = 0.46, overall fit = 0.498, Table S2) showed that the main determinant of higher platelet count was higher CRP, whereas lower serum iron (0.283 of the overall fit), although significantly associated with higher platelet count (*p* = 0.044), ranked fifth as predictor of platelets.

## 4. Discussion

In population studies, anemia and iron deficiency are often used interchangeably because of the high global prevalence of iron deficiency [2,8]. Accordingly, we expected to find that iron supplementation would be associated with decreased odds of anemia, low ferritin, and/or low serum iron. We also expected that lower ferritin, lower serum iron, and/or greater sTfR would explain a high proportion of the anemia model. However, these associations were not supported by our findings as iron status indicators were differentially associated with biomarkers of infection and inflammation, and importantly, with other nutrient deficiencies.

Our exploratory study identified the following key findings. (1) Anemia of malnutrition and anemia associated with iron deficiency predominated over anemia of inflammation. (2) Low plasma volume was present and likely contributed to an underestimation of anemia. (3) Iron deficiency co-existed with iron restriction due to inflammation, as evidenced by the association of higher CRP with higher ferritin, higher hepcidin, and low serum iron. (4) Low serum iron and hepcidin concentrations were both associated with inflammation and malnutrition. (5) Elevated sTfR was not associated with RBC indices, serum iron, ferritin, or hepcidin, but was associated with low protein status and vitamin A deficiency. (6) Higher maternal MNS intake and vitamin D concentrations were associated with higher LAZ but prolonged maternal iron supplementation was negatively associated with infant length, raising important questions about the efficacy of maternal iron supplementation strategies during lactation in MINDI populations.

### 4.1. Inflammation

#### 4.1.1. CRP and Iron Indicators

In studies of iron status, CRP is a widely used biomarker of inflammation during both an acute phase response and during a chronic low-grade inflammation associated with chronic infections or non-infectious conditions [63]. Currently, WHO defines inflammation as a CRP > 5 mg/L [17]. In our MINDI population, although higher CRP was associated with increased odds of low serum iron (<8.9 µmol/L), CRP did not enter models for anemia or elevated sTfR and had low dominance among the significant factors in linear regression models for both ferritin (fourth of four) and hepcidin (fourth of six). We were also able to show using predictive probability graphs that a CRP concentration of 10 mg/L corresponded to serum iron equal to 8.9 µmol/L. However, in our MINDI population, despite the high number of co-existing infections, very few mothers had CRP ≥ 10 mg/L (6%) and there was no evidence of T-helper (Th)1 or Th2 cytokines entering models for anemia or iron status indicators in this study. Previously, we had reported in early lactation that CRP was positively correlated with neutrophils, platelet counts, and plateletcrit, but negatively with number of eosinophils [25]. In this MINDI cohort, 27% of mothers had eosinophilia. Eosinophilia is indicative of a Th2 inflammatory response commonly associated with chronic helminth and fungal infections [64] which were endemic in this cohort [24]. Moreover, some Th-2 related infections have been associated with lower CRP in pregnancy and lactation [25], which likely reflects the weighted balance of Th1 vs Th2 immune responses and the down-regulation of Th-1 induced inflammation by Th2 cytokines. If overall inflammation was dampened, this might explain why CRP did not directly influence anemia or sTfR and why it occupied lower rank in dominance for serum iron and ferritin. Therefore, we concluded that in the MINDI cohort, although chronic low-grade inflammation may have a role in lowering circulating iron and increasing ferritin and hepcidin, inflammation was not a direct determinant of anemia or elevated sTfR.

#### 4.1.2. Platelets

Of interest in this remote setting was the fact that platelets, an indicator easily measured at the local hospital, had first rank dominance in the model for low serum iron, with values > 415,000/mm^3^, which is the upper value for normal platelets in non-pregnant women [39], and that corresponded to serum iron of 8.9 µmol/L. Thus, platelets may be an interesting alternative indicator of inflammation, as observed previously in adult [65] and obstetric populations [66].

#### 4.1.3. Inflammation and Ferritin

Different approaches for adjusting an increase in ferritin for inflammation have been used in women of reproductive age, but pregnant and lactating women have not been studied. Approaches have included correcting ferritin concentrations through equations that adjust for inflammation (CRP and/or alpha-1 glycoprotein) and the presence of malaria, or by excluding participants with elevated CRP on the assumption that inflammation increases ferritin [67]. In our case, because of the coexistence of Th1 and Th2 infections but no malaria [25], we did not apply these approaches. WHO recommends the cut-off of < 70 µg/L for low ferritin in populations with infection or inflammation [38]. However, in our MINDI cohort, 94% of lactating women including those with the highest CRP concentrations had ferritin values < 70 µg/L, suggesting that in the MINDI cohort, this cut-off would overestimate iron deficiency. On the other hand, application of the WHO cut-off for ferritin in the absence of inflammation (<15 ug/L) would indicate that 26.3% of women were iron deficient, an underestimate based on our evidence that 45.5% of women had low serum iron. Of note, the ferritin cut-off of < 30 µg/L used by the Panamanian Ministry of Health identified 63.6% of women with low iron stores. Given that serum iron represents the constantly changing circulating pool of iron in transit from one location to another [15], our data also suggest that, despite its negative association with inflammation, serum iron might be a more appropriate indicator of iron status under MINDI conditions.

#### 4.1.4. Inflammation and Anemia

Despite the presence of iron restriction due to inflammation, our model for anemia did not support the presence of anemia of inflammation. Anemia of inflammation is characterized by elevated hepcidin, elevated ferritin, and low serum iron [68], and hepcidin has been proposed as a biomarker to identify anemia of inflammation [69,70]. In contrast, in our population, these three indicators were lower in anemic mothers, which is characteristic of iron deficiency anemia [68]. However, in the hepcidin model, hemoglobin and serum iron ranked first and second in the dominance analysis and explained 43% of the model fit, indicating that hepcidin concentrations were associated with iron deficiency more than with inflammation. In support of this, hepcidin had been proposed as an iron status indicator during lactation [71], and it has been associated with iron deficiency rather than inflammation in post-partum women from the US [71], an observation that aligns with our findings.

### 4.2. Indicators of Malnutrition were Associated with Hepcidin, Serum Iron and sTfR

In addition to associations with inflammation, hepcidin, serum iron, and sTfR were associated with malnutrition. Both lower intakes of animal-source foods/week and lower concentrations of IGF-1, an indicator of nutritional status [37], were associated with higher hepcidin. Hepcidin has been reported to be a gluconeogenic sensor during starvation in mice [72]. In healthy humans, hepcidin was found to increase approximately 3-fold after prolonged fasting, and to decrease by 65% with the administration of growth hormone [73]. We also found evidence that higher IGF-1 decreased the odds of low serum iron in support of an earlier report of low serum iron concentrations in populations of lactating women with poor nutritional status [19].

On the other hand, elevated sTfR, a known indicator of erythropoiesis response following iron deficiency [74], was not associated with iron status indicators or inflammation indicators, but with the retinol/RBP molar ratio, which ranked first in dominance. It is known that both vitamin A [75] and protein [76] have a role in erythropoiesis. In this MINDI population, higher RBP, an indicator of both protein [37] and of vitamin A status [77], was associated with lowered odds of an elevated sTfR, suggesting that both nutrients alter the erythropoietic response. This suggestion requires further investigation.

### 4.3. Evidence of Anemia of Malnutrition during Lactation

Malnutrition has been associated with anemia during lactation where it coexists with low BMI [78], with a negative energy balance in working lactating women [79], and with the seasonal availability of food [80]. We saw evidence of this in the Ngäbe indigenous population, where among indicators associated with nutritional status, vitamin A ranked second and was followed by IGF-1 and plasma volume. Moreover, both higher IGF-1 and vitamin A were associated with lower odds of anemia. IGF-1 is recognized as an anabolic hormone having functions similar to insulin with regards to peripheral glucose and amino acid uptake, promoting energy storage; therefore, its release depends on sufficient substrate availability, whereas fasting and caloric restriction decrease IGF-1 [81]. Others have observed an association between low IGF-1 with anemia in adults, which authors explained by the role of IGF-1 in promoting RBC growth and differentiation, or by a higher IGF-1 reducing concentrations of pro-inflammatory cytokines that would block erythropoiesis [82]. Our associations of lower IGF-1 with increased odds of anemia, increased odds of low serum iron, and elevated sTfR, provide evidence that energy deficits contributed to anemia and low iron status in our MINDI cohort.

Unexpectedly, plasma volume, calculated using Nadler’s formula [53] that incorporates maternal weight and height, ranked as the fourth dominant factor, and was negatively associated with increasing the odds of anemia in lactating mothers. There is evidence that low plasma volume is an indicator of poor nutritional status in children [83] and adolescents [84]. Moreover, as plasma volume was low (<2.2 L) in 79.8% of lactating mothers, our findings suggest that anemia was most likely underdiagnosed. Others have emphasized the impact of low plasma volume from hypo-hydration or intra-vascular volume depletion impacting the diagnosis of anemia in tropical settings [85], and hypohydration has been reported in lactating women living from Indonesia [86] surrounded by scarcity of clean water [87], but to our knowledge, ours is the first study showing a possible impact of low plasma volume on the interpretation of hematological indicators under MINDI conditions.

### 4.4. Nutrient Supplementation during Lactation

In lactation, WHO recommends supplementation with iron alone or in combination with folic acid for 6–12 weeks after delivery, independently of lactation status, to reduce the risk of maternal anemia, particularly in areas where gestational anemia is a public health problem [88]. In the MINDI cohort, most lactating women were supplemented with iron and MNS beginning in pregnancy. The general rationale for iron supplementation during pregnancy is that pregnant women who are anemic in the first two trimesters are at higher risk of preterm delivery, low birthweight, infant mortality, and iron deficiency in the newborn [89]. However, a recent review found that iron supplementation during pregnancy had a limited effect on iron status of the offspring [90], and it is known that maternal iron status does not influence the iron content of breastmilk [91]. Moreover, the beneficial role of universal iron supplementation in iron-replete pregnant women and children has been questioned in developing countries given the possibility of iron supplementation increasing inflammation [92]. In our study, iron supplementation was not associated with anemia, ferritin, or serum iron. However, a longer duration of iron supplementation was associated with higher hepcidin, indicating that longer exposure to iron supplementation under MINDI conditions may increase the intracellularly sequestered iron pool, making it unavailable for erythropoiesis [93].

To address vitamin A deficiency, Panamanian women are given 200,000 IU of vitamin A after delivery. However, based on the retinol/RBP molar ratio that measures retinol saturation as an indicator of vitamin A deficiency post-partum [45,46], we found that protein deficiency may be an overlooked issue in this population and might help in explaining the lack of correlation between vitamin A and RBP. Under conditions of normal protein status, vitamin A concentrations determine vitamin A saturation with a normal retinol/RBP ratio of 1:1 [46], and low saturation indicates low retinol stores. However, protein malnutrition is known to decrease the synthesis of RBP and the affinity of retinol for RBP, leading to higher retinol saturation [94]. As the intake of high-quality protein is crucial for the conversion of pro-vitamin A into its active form [77], high concentrations of unbound vitamin A may occur in the absence of high-quality protein [49] and could theoretically lead to retinol toxicity. However, studies on the effect of vitamin A supplementation under conditions of protein deficiency are lacking. In our lactating women, the retinol/RBP molar ratio was <0.8 in 44% of mothers indicating vitamin A deficiency, but the ratio exceeded 1 in 34% of women, indicating the likelihood of unbound, and potentially toxic, vitamin A. Moreover, we found that a higher retinol/RBP ratio increased the likelihood that sTfr would be >8.3 mg/L, indicating a need for iron supplementation when in fact the mothers may require more protein. In this MINDI cohort, 36.4% of mothers had low RBP that was also accompanied by low hemoglobin, neutrophils, monocytes, and IL-13 compared with protein-sufficient women. Intake of high-quality protein is also crucial for the conversion of pro-vitamin A into its active form [77], and protein malnutrition is known to decrease the synthesis of RBP, which will saturate to its limit after a high dose of vitamin A [94]. Therefore, our findings suggest that caution is needed when choosing to supplement with a megadose of vitamin A without considering a need to also increase dietary intake of animal-source foods.

### 4.5. Maternal Determinants of Infant Anthropometry

Our study uncovered evidence that higher maternal plasma volume and lower platelet indices were positively associated with infant anthropometric indices. Specifically, increased maternal plasma volume was associated with higher WAZ and LAZ and ranked first in dominance for both. Furthermore, among the inflammation markers, higher platelet indices but not CRP were negatively associated with all three anthropometric indices: platelet counts with WAZ and LAZ and plateletcrit with HCAZ. These findings suggest an extension of the previously observed negative impact of maternal inflammation on lower symphysis-fundal height during pregnancy [95], into early lactation.

Maternal nutrient intakes and nutritional status during lactation were also associated with early infant growth. Maternal intake of animal-source foods ranked second in dominance, where it was positively associated with WAZ. Others have linked the provision of animal-source foods with growth [96], but this is the first study to show that maternal intake of protein during the first 6 months postpartum might be important for early infant growth. In our study, we also found that higher maternal intake of MNS, containing protein, energy, and micronutrients [97], was associated with higher LAZ, an important observation given that 16% of infants ≤ 7 mo were already stunted. Given the increasing prevalence of stunting during the first 2 years in the Ngäbe population [98], strengthening government-sponsored MNS supplementation might lessen the impact of maternal malnutrition during lactation on early infant growth by increasing the intake of protein and a range of micronutrients.

In contrast to MNS supplementation, the provision of iron tablets had a decidedly negative association with linear growth. There is growing concern about a universal iron supplement in developing countries, given an increased risk of perinatal maternal and/or infant infections due to a higher availability of iron to pathogens [99]. In Panama, at the time of this study, 60 mgs of ferrous fumarate were prescribed to pregnant women from the second trimester through to 3 months post-partum [100]. Women were also prescribed iron beyond this period if presenting with anemia. Our findings provide evidence that the duration of iron supplementation under MINDI conditions may be problematic and that universal iron supplementation in early lactation has negative consequences for the infant and may contribute to stunting.

We also uncovered evidence that the high prevalence of maternal vitamin D deficiency in lactation was contributing to impaired linear growth as higher maternal serum 25OH D3 was positively associated with LAZ in the first 6 months postpartum. Presently, there is no consensus regarding vitamin D supplementation during lactation. On one hand, it is known that vitamin D concentrations in milk are directly related to maternal vitamin D status [101] and that maternal vitamin D deficiency has been associated with small for gestational age [102]. However, among recent randomized, double-blind, placebo-controlled trials of maternal vitamin D supplementation, one in Bangladesh found no effect of maternal supplementation on infant LAZ, although infants were not exclusively breastfed [103], and a second in Arab mothers found that maternal supplementation was able to achieve normal vitamin D concentrations in exclusively breastfed infants [104]. In our exclusively and predominantly breastfeeding mothers, our model of LAZ showed that vitamin D and MNS ranked fourth and fifth, respectively, and were positively associated with LAZ, following a positive association of plasma volume (first), and negative associations of longer duration of maternal iron supplementation (second) and inflammation indicated by maternal platelet (counts per mm^3^) (third), with LAZ. Thus, our results show that while vitamin D may be needed to improve linear growth, it may not be sufficient to overcome the negative impact of excess iron or the presence of maternal inflammation and/or chronic infections, which need to be considered as important determinants of stunting. Moreover, as 69% of lactating mothers were vitamin D deficient, our findings also support the need to consider a recommendation for vitamin D supplementation to mothers and/or infants as part of a public health policy, as vitamin D is not only associated with infant growth but with immune function and health [105].

## 5. Conclusions

In conclusion, we found a high prevalence of anemia associated with malnutrition in lactating mothers in the Ngäbe Bugle indigenous region. Anemia of malnutrition was multifactorial and included deficiencies in iron, vitamin A, and protein-energy malnutrition, coexisting with infections and inflammation. Of concern was that the duration of iron supplementation throughout pregnancy and lactation was associated with impaired linear growth within the first 6 months. Importantly, indicators of better maternal nutrition in early lactation and of less inflammation using platelet indices instead of CRP were associated with improved infant anthropometry.

## 6. Strengths and Limitations

The strengths of the study include an in-depth exploration of a wide range of parameters employing strict variable selection using bootstrapping and MFP modeling that uncovered meaningful associations with iron status indicators and infant growth during early lactation in our MINDI cohort of lactating women. We explicitly included several biomarkers of inflammation in regression models to control all iron indicators for inflammation, rather than creating new adjusted variables using CRP alone for only ferritin. We also explored the associations with serum iron, a marker of iron status not often included in studies from developing countries. This highlighted the importance of its specificity over classic iron status indicators in our study population. We also were able to include hepcidin, which not only emerged as a marker of iron status but of malnutrition.

This was a cross sectional study that precludes causation. However, as an observational study with both a prospective and retrospective elements, we were able to control for confounders and to assess multiple exposures and several understudied outcomes. Sample size might be criticized as a limitation, however, the high prevalence of anemia (>40%) provided enough power to study this outcome given that we were able to recruit 99 women with the assistance of the Ministry of Health, accounting for nearly all women who had delivered in the past 6 months. However, to be cautious, we also chose a rigorous statistical analysis framework, recognizing that this is the first study to shed light on the multifactorial causes of anemia and understanding that other confirmatory studies will be required. We also acknowledge that in an ideal context, post-partum women would be best studied at similar weeks post-partum but this was impractical in our remote setting. Regarding supplementation, we assumed that all women were supplemented with vitamin A close to delivery by the Ministry of Health according to their protocols, but information on vitamin A supplementation of women delivering at home was not collected. Confirmation of supplementation was self-reported at follow up visits by the research team where the dosage of both iron tablets and MNS and duration of iron intakes were recorded.

## Figures and Tables

**Table 1:** Population characteristics of indigenous lactating women from Panama and their infants.

Variable	Mean ± SD or Median (Min, Max)
Maternal Characteristics	
^3^ Age, years	23 (14–42)
^3^ Parity, #	3 (1–11)
^3^ Weeks post-partum	12 (1–28)
Home delivery, %	42.4
Wood smoke, %	93.9
Fieldwork, %	41.4
Taking iron tablets, %	92.9
^4^ Weeks taking iron during pregnancy	10 (0–39)
^5^ Weeks taking iron during lactation	9 (0–28)
Taking multiple nutrient supplements, %	39.4
BMI, kg/m^2^	25.6 ± 3.02
<18.5, %	0
18.5–24.9, %	48.5
25.0–29.9, %	43.4
30.0–34.9, %	8.1
**Reported Weekly Intakes**	
^3^ Animal-source foods, %	2 (0–13)
^3^ Orange-red fruits & vegetables, %	1 (0–14)
^3^ Green leafy vegetables, %	1 (0–8)
**Maternal Infections**	
Caries, %	18.2
Scabies, %	8.1
^1^ Urinary leukocyte esterase (+), %	76.0
Vaginal microorganisms	
^1^ *Lactobacillus*, %	26.6
^2^ *Bacteroides/Gardnerella, %*	97.5
^1^ *Mobiluncus, %*	87.3
^1^ *Trichomonas* *, %*	91.1
Yeast, %	11.4
^2^ *Diplococcus, %*	31.6
**Infant Characteristics**	
^1^ Gestational age at birth	40 (32–42)
Preterm delivery (<37 weeks), %	5.0
^3^ Age,weeks	12 (1–28)
Exclusive breastfeeding, %	84.8%
Predominantly breastfeeding, %	15.2%
HCAZ	0.05 ± 1.34
HCAZ < −2 SD	5.0%
LAZ	−0.36 ± 1.60
LAZ < −2 SD	16.3%
WAZ	0.31 ± 1.48
WAZ < −2 SD	9.1%

Sample size = 99 (^1^ *n* = 94, ^2^ *n* = 79). Values are means ± standard deviation (SD) if normally distributed, median (min-max) if not normally distributed, or percentages (%) if binary, unless otherwise specified. ^3^ Interquartile range (25th and 75th percentiles) for not-normally distributed data: maternal age (20, 30 years); parity (2, 5); weeks post-partum (4, 16); gestational age at birth (40, 40 weeks); infant age (4, 16 weeks); weekly intakes of animal-source foods (1–4), orange/red fruits and vegetables (0–2), green leafy vegetables (0–3). ^4^ Weeks on iron during pregnancy = positive values of (weeks on iron − weeks post-partum); ^5^ weeks on iron during lactation = total weeks on iron − weeks on iron during pregnancy. Abbreviations: BMI, body-mass index; HCAZ, head circumference for age Z-score; LAZ, length for age Z-score; SD, standard deviation; WAZ, weight for age Z-score.

**Table 2:** Biochemical indicators of nutritional status, iron status, immunity, and inflammation in indigenous lactating women from Panama.

Variable	Mean ± SD; %; Median (Min, Max)	IQR
Nutritional Biomarkers		
Folic acid, nmol/L	11.9 (5.4–35.9)	8.9, 16.2
Folic acid < 10 nmol/L	31.3%	
Vitamin B_12_, pmol/L	153 (82–505)	128, 190
B_12_ < 150 pmol/L	46.5%	
Vitamin D, nmol/L	42.2 (14.4–75.0)	32.7, 52.5
Vitamin D < 50 nmol/L	68.7%	
Vitamin A, µmol/L	1.4, (0.6–2.9)	1.1, 1.8
Vitamin A < 1.05 µmol/L	18.6%	
Retinol binding protein (RPB), mg/L	42.6 (3.4–169.3)	25.6, 57.7
RBP < 30 mg/L	36.4%	
Retinol/RBP molar ratio	0.9 (0.1–8.8)	0.5, 1.1
Vitamin A saturation < 0.8	44.4%	
Vitamin A saturation > 1	34.2%	
IGF-1, ng/mL	31.0 (14.5–65.2)	26.1, 34.6
Calculated plasma volume, L	2.0 (1.6–3.1)	1.8, 2.1
Serum iron, µmol/L	10.2 (2.8–31.0)	7.3, 14.1
Serum iron < 8.9 µmol/L	45.4%	
Ferritin, µg/L	23.5 (1.7–129.1)	13.9, 36.1
Ferritin < 70 µg/L	93.9%	
Ferritin < 30 µg/L	63.6%	
Ferritin < 15 µg/L	26.3%	
sTfR, mg/L	7.9 (2.3–20.8)	6.2, 9.8
sTfR > 8.3 mg/L	41.4%	
Hepcidin, µg/L	15.6 (5.1–56.8)	11.6, 20.8
**Blood Cell Indices**		
Hemoglobin, g/L	117.5 ± 10.8	
RBC × 10^6^/mm^3^	4.1 ± 0.37	
Hematocrit	37.8 (24.3–44.3)	35.7, 39.7
Hematocrit < 36%	27.3%	
MCV, fL	92.8 ± 5.1	
MCH, pg	29.0 ± 1.8	
MCHC, g/L	31.3 ± 1.1	
^1^ RDW-CV	14 (12–18)	13, 14
WBC × 10^3^/mm^3^	8.2 ± 1.9	
Neutrophils × 10^3^/mm^3^	4.2 (1.8–8.8)	3.5, 5.2
Lymphocytes × 10^3^/mm^3^	2.4 ± 0.6	
Monocytes × 10^3^/mm^3^	0.36 (0.19–0.86)	0.32, 0.44
Eosinophils × 10^3^/mm^3^	0.61 (0.07–4.26)	0.34, 0.91
Eosinophils > 0.6 × 10^3^/mm^3^	50.5%	
Basophils × 10^3^/mm^3^	0.04 (0.01–0.12)	0.03, 0.06
Platelets × 10^3^/mm^3^	306 (180–638)	250, 363
Platelets > 415 × 10^3^/mm^3^	16.2%	
^1^ Plateletcrit (%)	26.9 (17.2–52.0)	23.5, 31.5
**CRP and Cytokines, pg/mL**		
CRP, mg/L	1.3 (0.1–16.9)	0.5, 3.7
CRP > 5 mg/L	20.2%	
CRP > 10 mg/L	6.1%	
IL-1β	0.02 (0.01–38.6)	0.02, 1.3
IL-4	2.2 (0.0–37.0)	0.2, 4.3
IL-6	1.6 (1.6–88.9)	1.6, 1.6
IL-10	0.3 (0.0–23.9)	0.1, 1.3
IL-12	1.3 (0.0–42.8)	0.1, 5.7
IL-13	0.9 (0.1–15.7)	0.6, 1.6
IL-17	0.9 (0.1–87.1)	0.5, 3.5
IFN-γ	1.6 (0.05–223.3)	0.05, 3.5
TNF-α	4.7 (0.04–24.4)	1.3, 8.2
MCP-1	315.4 (46.0–586.0)	256.5, 391.1

Sample size = 99 (^1^ *n* = 97); IQR (interquartile range) corresponds to the 25th and 75th percentiles. Values are means ± standard deviation (SD) if normally distributed, median (min-max and interquartile range) if not normally distributed, or percentages (%) if binary, unless otherwise specified. Abbreviations: CRP, C-reactive protein; IFN, interferon; IGF-1, insulin-like growth factor; IL, interleukin; MCH, mean corpuscular hemoglobin; MCHC, mean corpuscular hemoglobin concentration; MCP-1, monocyte chemoattractant protein-1; MCV, mean corpuscular volume; RBC, red blood cells; RBP, retinol-binding protein; RDW-CV, red cell deviation width—coefficient of variation; SD, standard deviation; sTfR, serum transferrin receptor; TNF, tumor necrosis factor; WBC, white blood cells.

**Table 3:** Comparison of lactating indigenous women and their infants based on maternal anemia ^1^.

	Presence of Anemia (*n* = 44)		Absence of Anemia (*n* = 55)	
Variables				
Maternal Characteristics	Mean ± SD; %; Median (Min, Max)	IQR	Mean ± SD; %; Median (Min, Max)	IQR
Weeks post-partum	11.5 (2 − 26)	7, 16	12 (1–28)	4, 17
BMI, kg/m^2^	25.2 ± 0.4		25.9 ± 0.4	
Supplementation				
Iron tablets	90.9%		94.5%	
Number of wks taking iron in pregnancy	6.5 (0–39)	0.5, 21	11 (0–37)	4, 23
Number of wks taking iron in lactation	8 (0–23)	3, 13	10 (0–28)	4, 17
Number of tablets × 600 mg/d	1 (0–4)	1, 2	1 (0–3)	1, 1
Multiple nutrient supplements	29.5%		47.3%	
**Intakes ≥ 1 Portion/wk**				
Animal-source foods	79.5%		81.8%	
Orange-red fruits & vegetables	68.1%		54.3%	
Green leafy vegetables	54.5%		56.4%	
**Nutritional Biomarkers**				
Folic acid, nmol/L	11.2 (6.6–31.9)	8.4, 14.7	12.1 (5.4–35.9)	10.2, 16.2
<10 nmol/L	* 43.2%		21.8%	
Vitamin B_12_, pmol/L	152 (87–505)	131, 186.5	157 (82–399)	123, 200
<150 pmol/L	45.4%		47.3%	
Vitamin D, nmol/L	39.4 (14.4–69.7)	31.0, 48.1	44.7 (17.8–75.0)	34.2, 57.9
<50 nmol/L	* 79.5%		60.0%	
Vitamin A, µmol/L	** 1.3 (0.6–2.2)	1.0, 1.5	1.6 (0.6–2.9)	1.3, 2.0
<1.05 µmol/L	25.0%		13.2%	
Retinol Binding Protein, mg/L	31.2 (3.4–169.3)	23.8, 55.3	45.4 (5.4–118.7)	28.2, 62.0
<30 mg/L	45.4%		29.1%	
Retinol/RBP molar ratio	0.9 (0.1–8.8)	0.4, 1.2	0.8 (0.1–7.7)	0.5, 1.1
<0.8	43.2%		45.4%	
>1	40.9%		28.3%	
IGF-1, ng/Ml	30.5 (14.5–65.2)	21.9, 33.9	31.8 (21.5–61.9)	28.4, 36.0
Serum iron, µmol/L	*** 7.8 (2.8–31.0)	6.0, 11.2	12.1 (3.6–23.0)	8.3, 15.2
<8.9 µmol/L	* 59.1%		34.5%	
Ferritin, µg/L	* 17.8 (1.7–127.9)	12.1, 33.4	28.3 (5.9–129.1)	17.1, 42.1
<70 µg/L	95.4%		92.7%	
<30 µg/L	72.7%		56.4%	
<15 µg/L	34.1%		20.0%	
sTfR, mg/L	27.8 (2.3–20.8)	5.9, 9.7	7.9 (2.3–15.8)	6.4, 10.1
>8.3 mg/L	37.7%		45.7%	
Hepcidin, µg/L	* 14.6 (5.1–56.8)	11.3, 20.0	16.6 (5.4–53.7)	12.3, 21.5
**Blood Cell Indices**				
Hemoglobin, g/L	*** 108.7 ± 8.2		124.6 ± 6.8	
RBC × 10^6^/mm^3^	*** 3.8 ± 0.4		4.2 ± 0.3	
Hematocrit	*** 35.7 (24.3–38.8)	33.7, 37.1	39.6 (34.3–44.3)	38.4, 41.0
MCV, fL	* 91.5 ± 5.5		93.7 ± 4.5	
MCH, pg	** 28.5 ± 2.0		29.5 ± 1.5	
MCHC, g/L	31.1 ± 1.3		31.5 ± 0.9	
^1^ RDW-CV, %	14 (12–18)	13, 15	14 (12–16)	13, 14
WBC × 10^3^/mm^3^	8.23 ± 2.0		8.20 ± 1.77	
Neutrophils, × 10^3^/mm^3^	4.14 (2.47–8.41)	3.52, 5.23	4.21 (1.80–8.83)	3.40, 5.24
Lymphocytes, × 10^3^/mm^3^	2.47 ± 0.66		2.44 ± 0.60	
Monocytes, × 10^3^/mm^3^	0.37 (0.22–0.63)	0.33, 0.45	0.36 (0.19–0.86)	0.31, 0.44
Eosinophils, × 10^3^/mm^3^	0.50 (0.09–2.04)	0.34, 0.90	0.65 (0.07–4.26)	0.34, 1.17
Basophils, × 10^3^/mm^3^	0.04 (0.01–0.12)	0.03, 0.06	0.04 (0.01–0.12)	0.03, 0.06
Platelets × 10^3^/mm^3^	317 (214–638)	273, 407	296 (180–619)	233, 343
^1^ Plateletcrit (%)	* 27.1 (19.7–44.1)	25.5, 35.0	26.6 (17.2–52.0)	21.8, 30.1
**CRP and Cytokines, pg/mL**				
CRP, mg/L	1.2 (0.1–16.9)	0.4, 4.1	1.4 (0.1–14.0)	0.7, 3.4
IL-1β	0.5 (0.01–7.4)	0.02, 1.3	0.02 (0.01–38.6)	0.01, 1.7
IL-4	2.6 (0.0–37.0)	0.2, 3.9	1.5 (0.0–34.7)	0.2, 4.3
IL-6	1.6 (1.6–88.9)	1.6, 1.6	1.6 (1.6–51.9)	1.6, 1.6
IL-10	0.3 (0.0–19.9	0.1, 0.6	0.3 (0.0–23.9)	0.0, 1.6
IL-12	1.8 (0.0–20.8)	0.1, 5.5	0.8 (0.0–42.8)	0.1, 5.3
IL-13	0.9 (0.1–15.7)	0.6, 1.3	0.9 (0.1–8.0)	0.9, 1.6
IL-17	0.7 (0.1–87.1)	0.5, 1.6	1.0 (0.1–69.6)	0.6, 2.0
IFN-γ	1.6 (0.05–62.1)	0.05, 2.8	1.7 (0.05–223.3	0.05, 5.2
TNF-α	5.4 (0.04–23.1)	1.2, 10.0	4.4 (0.04–24.4)	1.3, 7.9
MCP-1	314.8 (46.0–586.0)	261.0, 384.4	317.8 (46.0–586.0)	238.0, 391.1
**Infant Anthropometry**				
HCAZ	−0.01 ± 1.14		0.11 ± 1.49	
LAZ	−0.39 ± 1.54		−0.34 ± 1.66	
WAZ	0.06 ± 1.19		0.50 ± 1.66	

^1^ Anemia was defined as hemoglobin < 110 g/L if ≤ 8 weeks post-partum, and < 120 g/L if > 8 weeks post-Partum. Sample size = 99 (^1^ *n* = 97). Values are means ± standard deviation (SD) if normally distributed, median (min, max, interquartile range) if not normally distributed, or percentages (%) if binary. *t*-test or Kruskal–Wallis test for continuous variables; Chi ^2^ Test for proportions (or Fisher’s Exact Test if any cell ≤ 5). * *p* < 0.05, ** *p* < 0.01, *** *p* < 0.001. Abbreviations: BMI, body mass index; CRP, C-reactive protein; HCAZ, head circumference for age Z-score; Hb, hemoglobin; IFN, interferon; IGF-1, insulin-like growth factor; IL, interleukin; IQR: interquartile range; LAZ, length for age Z-score; MCH, mean corpuscular hemoglobin; MCHC, mean corpuscular hemoglobin concentration; MCP-1, monocyte chemoattractant protein-1; MCV, mean corpuscular volume; RBC, red blood cells; RBP, retinol-binding protein; RDW-CV, red cell deviation width—coefficient of variation; SD, standard deviation; sTfR, serum transferrin receptor; TNF, tumor necrosis factor; WAZ, weight for age Z-score; WBC, white blood cells.

**Table 4:** MFP logistic regression models for (A) anemia, (B) serum transferrin receptor (sTfR) > 8.3 mg/L, and (C) serum iron < 8.9 µmol/L iron, with dominance analysis indicating ranking of predictors.

Model and Variables	MFP Logistic Regression Models			Dominance Analysis	
(A)					
Anemia ^1^	OR ± SE	*p*-Value	95% CI	Rank	Standardized Dominance
t-Serum iron, µmol/L *	0.86 ± 0.05	0.005	0.77, 0.95	1	0.319
t-Vitamin A, µmol/L	0.18 ± 0.11	0.006	0.05, 0.61	2	0.286
t-Log IGF-1, ng/mL	0.08 0.09	0.022	0.43, 1.15	3	0.195
t-Plasma volume, dL *	1.44 ± 0.20	0.010	1.09, 1.90	4	0.188
t-Weeks post-partum	1.04 ± 0.04	0.329	0.96, 1.11	5	0.011
Constant	0.71 0.18	0.166	0.43, 1.15	Overall fit statistic = 0.227	
**(B)**					
**sTfR > 8.3 mg/L ^2^**	**OR ± SE**	***p*-Value**	**95% CI**	**Rank**	**Standardized Dominance**
t-Retinol/RBP molar ratio *	2.69 ± 0.99	0.007	1.30, 5.57	1	0.495
Home delivery (0 = no, 1 = yes)	2.32 ± 1.15	0.089	0.88, 6.12	2	0.172
t-IGF-1, ng/mL	1.05 ± 0.03	0.091	0.99, 1.11	3	0.166
t-Field work, h/d	1.15 ± 0.10	0.111	0.97, 1.37	4	0.141
t-Weeks post-partum	1.03 ± 0.03	0.433	0.96, 1.09	5	0.026
Constant	0.40 ± 0.13	0.007	0.21, 0.78	Overall fit statistic = 0.149	
**(C)**					
**Serum Iron < 8.9 µmol/L ^3^**	**OR ± SE**	***p*-Value**	**95% CI**	**Rank**	**Standardized Dominance**
t-Platelets × 10^5^/mm^3^	2.39 ± 0.83	0.012	1.21, 4.72	1	0.236
t-Ferritin, µg/L	0.95 ± 0.01	0.004	0.92, 0.98	2	0.190
t-CRP, mg/L	1.29 ± 0.14	0.019	1.04, 1.60	3	0.182
t-IGF-1, ng/mL	0.91 ± 0.04	0.031	0.83, 0.99	4	0.161
t-NLR	2.00 ± 0.78	0.076	0.93, 4.32	5	0.100
t-Green/leafy vegetables, portions/wk	0.80 ± 0.10	0.074	0.64, 1.02	6	0.089
t-Weeks post-partum	1.09 ± 0.05	0.057	0.99, 1.19	7	0.041
Constant	0.77 ± 0.22	0.353	0.44, 1.33	Overall fit statistic = 0.357	

* Winsorized variables: extreme high observations of serum iron (*n* = 1), plasma volume (*n* = 1), and retinol/RBP molar ratio (*n* = 3) were replaced. ^1^ Anemia model (*p* < 0.0001, pseudo R2 = 0.23, AUC = 0.79, *n* = 94, VIF = 1.02, condition number = 1.22). Variables with ≥ 500 bootstrap repetitions but excluded by MFP Process: maternal age, BMI category, intake of MNS, platelet count, MCV, vitamin D (nmol/L). Transformation of covariates (t): Weeks post-partum = weeks post-partum − 11.77659574; plasma volume (dL) = plasma volume − 19.87454379; serum iron (µmol/L) = serum iron – 10.66595738; vitamin A = vitamin A − 1.481,764,884; log insulin-growth factor-1 (IGF-1) = log IGF-1 − 3.415743564. ^2^ sTfR model (*p* = 0.0027, pseudo R^2^ = 0.15, AUC = 0.76, *n* = 92, VIF = 1.04, condition number = 2.27). Variables with ≥ 500 bootstrap repetitions but excluded by MFP process: wood smoke, months on iron supplementation, platelet distribution width. Transformation of covariates (t): weeks post-partum = weeks post-partum − 11.77173913; field work = fieldwork/d − 1.97826087; retinol/RBP molar ratio = retinol/RBP− 0.9917081245; insulin growth factor-1 = IGF-1 − 31.46847824. **^3^** Serum Iron model (*p* < 0.0001, pseudo R^2^ = 0.36, AUC= 0.87, *n* = 94, VIF = 1.25, condition number = 2.27). Variables with ≥ 500 bootstrap repetitions but by MFP process: RDW-CV, hemoglobin, hematocrit, IL-4. Transformation of covariates (t): weeks post-partum = weeks post-partum − 11.77659574; green/leafy vegetables = portions of green/leafy vegetables − 1.872340426; platelets = platelet count − 3.265106383; neutrophil/lymphocyte ratio (NLR) = NLR − 1.954397652; C-reactive protein (CRP) = CRP − 2.95531915; ferritin = ferritin − 30.2882978; insulin-like growth factor-1 (IGF-1) = IGF-1 − 31.5585106.

**Table 5:** MFP linear regression models for (A) ferritin including hepcidin, (B), ferritin excluding hepcidin, (C) hepcidin including ferritin, and (D) hepcidin excluding ferritin, with dominance analysis indicating ranking of predictors.

Model and Variables	MFP Linear Regression Models				Dominance Analysis	
(A)						
Log Ferritin (µg/L) with Hepcidin ^1^	Coeff ± SE	95% CI	β	*p*-Value	Ranking	Standardized Dominance
t-Hepcidin, µg/L	0.04 ± 0.007	0.03, 0.06	0.47	<0.0001	1	0.450
t-Serum iron, µmol/L	0.04 ± 0.01	0.01, 0.07	0.22	0.009	2	0.152
t-Hemoglobin, g/L	0.02 ± 0.01	0.004, 0.03	0.21	0.011	3	0.128
t-sTfR, mg/L	−0.07 ± 0.02	−0.10, −0.03	−0.25	0.001	4	0.126
t-CRP, mg/L	0.06 ± 0.02	0.02, 0.09	0.24	0.004	5	0.083
t-Weeks post-partum	0.0004 ± 0.01	−0.02, 0.02	0.003	0.964	6	0.011
Constant	3.10 ± 0.06	2.98, 3.22		<0.0001	Overall fit statistic = 0.589	
**(B)**						
**Log Ferritin (µg/L) without Hepcidin ^2^**	**Coeff ± SE**	**95% CI**	**β**	***p*** **-Value**	**Ranking**	**Standardized Dominance**
t-Serum iron, µmol/L	0.06 ± 0.02	0.03, 0.09	0.35	<0.0001	1	0.307
t-Hematocrit, %*	0.08 ± 0.02	0.03, 0.13	0.29	0.002	2	0.224
t-sTfr, mg/L	−0.08 ± 0.02	−0.12, −0.03	−0.29	0.001	3	0.217
t-CRP, mg/L	0.08 ± 0.02	0.04, 0.12	0.34	<0.0001	4	0.165
Caries, presence	−0.29 ± 0.19	−0.67, 0.08	−0.13	0.123	5	0.066
t-Weeks post-partum	−0.005 ± 0.01	−0.03, 0.02	−0.04	0.639	6	0.021
Constant	3.10 ± 0.07	2.96, 3.24		<0.0001	Overall fit statistic = 0.415	
**(C)**						
**Log Hepcidin (µg/L) with Ferritin ^3^**	**Coeff ± SE**	**95% CI**	**β**	***p*** **-Value**	**Ranking**	**Standardized Dominance**
t-Ferritin, µg/L	1.44 ± 0.16	1.12, 1.76	0.62	<0.0001	1	0.710
t-RBC × 10^6^/mm^3^	0.28 ± 0.09	0.09, 0.47	0.21	0.004	2	0.088
t-Animal-source foods, portions/wk	−0.03 ± 0.01	−0.05, −0.008	−0.19	0.008	3	0.085
t-IGF-1 pg/mL	−0.01 ± 0.004	−0.02, −0.003	−0.19	0.006	4	0.071
t-Months on iron supplements	0.02 ± 0.01	−0.003, 0.04	0.11	0.094	5	0.030
t-Weeks post-partum	−0.003 ± 0.005	−0.01, 0.006	−0.05	0.512	6	0.017
Constant	2.81 ± 0.03	2.75, 2.87		<0.0001	Overall fit statistic = 0.600	
**(D)**						
**Log Hepcidin (µg/L) without Ferritin ^4^**	**Coeff ± SE**	**95% CI**	**β**	***p*** **-Value**	**Ranking**	**Standardized Dominance**
t-Hemoglobin, g/L	0.02 ± 0.004	0.009, 0.02	0.38	<0.0001	1	0.220
t-Serum iron, µmol/L	0.03 ± 0.008	0.01, 0.04	0.32	0.001	2	0.211
t-Animal-source portions/wk	−0.05 ± 0.01	−0.07, −0.02	−0.32	<0.0001	3	0.171
t-CRP, mg/L	0.05 ± 0.01	0.02, 0.07	0.37	<0.0001	4	0.159
t-IGF-1, pg/mL	−0.01 ± 0.004	−0.02, −0.007	−0.29	0.001	5	0.140
t-Months on iron supplements	0.03 ± 0.01	0.006, 0.06	0.20	0.016	6	0.065
t-Weeks post-partum	−0.005 ± 0.006	−0.02, 0.007	−0.07	0.445	7	0.033
Constant	2.75 ± 0.04	2.68, 2.83		<0.0001	Overall fit statistic = 0.462	

* Winsorized variable: 1 extremely low observation of hematocrit was replaced. ^1^ Ferritin model with hepcidin (*p* < 0.0001, adj. R^2^ = 0.56, *n* = 94, VIF = 1.31, condition number = 2.03). Variables with ≥ 500 bootstrap repetitions by the MFP process: red blood cell and monocyte counts, hematocrit, mean platelet volume, IL-17, caries severity, urinary leukocyte esterase, folic acid, vitamin D. Transformation of covariates (t): weeks post-partum = weeks post-partum – 11.54255319; hemoglobin = hemoglobin − 117.8404255; hepcidin = hepcidin − 17.81840419; serum iron = serum iron − 10.76638289; serum transferrin receptor (sTfR) = sTfR − 8.220106373; C-reactive protein (CRP) = CRP − 2.997872347. ^2^ Ferritin model without hepcidin (*p* < 0.0001, adj. R^2^ = 0.38, *n*= 94, VIF = 1.22, condition number = 2.02). Variables with ≥ 500 bootstrap repetitions excluded by the MFP process: red blood cells, monocytes, hemoglobin, mean platelet volume, caries severity, urinary leukocyte esterase, folic acid, vitamin D. Transformation of covariates: weeks post-partum = weeks post-partum − 11.55208333; hematocrit = hematocrit − 37.64729174; serum iron = serum iron − 10.78906241; serum transferrin receptor (sTfR) = sTfR − 8.279687499; C-reactive protein (CRP) = CRP − 2.978125007. ^3^ Hepcidin model with ferritin (model *p* < 0.0001, adj. R^2^ = 0.57, *n* = 96, VIF = 1.08, condition number = 1.57). Variables with ≥ 500 bootstrap repetitions excluded by MFP process: diastolic blood pressure, hematocrit, hemoglobin, log-monocytes, CRP, IL-17, serum iron, folic acid, RBP. Transformation of covariates (t): weeks post-partum = weeks post-partum − 11.78125; months on iron supplements= months on iron − 5.0625; animal-source foods = animal-source foods − 3.125; red blood cell (RBC) = RBC − 4.071562529; ferritin = (ferritin/100) ^0.5^ − 0.5475703905; insulin-like growth factor-1 (IGF-1) = IGF-1 − 31.63958327. ^4^ Hepcidin model without ferritin (*p* < 0.0001, adj. R^2^ = 0.36, *n* = 96, VIF = 1.25, condition number = 2.18. Variables with ≥ 500 bootstrap repetitions excluded by MFP process: diastolic blood pressure, red blood cell count, hematocrit, log monocytes, IL-17, presence of caries, folic acid, retinol-binding protein. Transformations of covariates (t): weeks post-partum = weeks post-partum − 11.78125; months on iron supplements = months on iron supplements − 5.0625; animal-source foods = animal-source foods − 3.125; hemoglobin = hemoglobin − 117.78125; C-reactive protein, (CRP) = CRP − 2.936458339; serum iron = serum iron − 10.86260409; insulin-like growth factor-1 (IGF-1) = IGF-1 − 31.63958327.

**Table 6:** MFP linear regression models for infant anthropometric measurements.

Model and Variables	MPF Linear Regression Models				Dominance Analysis	
(A)						
HCAZ ^1^	Coeff ± SE	95% CI	β	*p*-Value	Ranking	Standardized Dominance
t-Parity, *n*	0.13 ± 0.05	0.02, 0.23	0.24	0.017	1	0.312
t-Plateletcrit, %	−0.04 ± 0.02	−0.08, −0.003	−0.22	0.034	2	0.212
t-Plasma volume, dL *	0.10 ± 0.06	−0.02, 0.22	0.16	0.103	3	0.176
t-Ferritin, µg/L	−0.01 ± 0.005	−0.02, 0.001	−0.17	0.079	4	0.120
t-Weeks post-partum	−0.04 ± 0.02	−0.07, 0.001	−0.20	0.059	5	0.107
t-GA at birth, wks	0.06 ± 0.07	−0.08, 0.98	0.08	0.416	6	0.073
Constant	−0.24 ± 0.12	−0.48, −3.35 × 10^−7^		0.050	Overall fit statistic = 0.201	
**(B)**						
**LAZ ^2^**	**Coeff ± SE**	**95% CI**	**β**	***p*** **-Value**	**Ranking**	**Standardized Dominance**
t-Plasma volume, mL	0.32 ± 0.06	0.19, 0.45	0.44	<0.0001	1	0.465
t-Months on iron supplements	−0.12 ± 0.05	−0.22, −0.02	−0.22	0.014	2	0.134
t-Platelets × 10^3^/mm^3^	−2.66 ± 1.33	−5.31, −0.02	−0.17	0.048	3	0.114
t-Vitamin D, nmol/L	0.02 ± 0.01	0.007, 0.04	0.23	0.008	4	0.108
t-MNS, tbsp/d	0.25 ± 0.09	0.07, 0.44	0.23	0.009	5	0.098
t-Gestational age, wks	0.14 ± 0.07	−0.01, 0.28	0.16	0.064	6	0.081
Constant	−0.67 ± 0.13	−0.93, −0.41		<0.0001	Overall fit statistic 0.379	
**(C)**						
**WAZ ^3^**	**Coeff ± SE**	**95% CI**	**β**	***p*** **-Value**	**Ranking**	**Standardized Dominance**
t-Plasma volume, L	0.19 ± 0.06	0.06, 0.32	0.27	0.004	1	0.273
t- Animal-source foods/portions/wk	0.12 ± 0.04	0.04, 0.21	0.26	0.005	2	0.239
t-Platelets × 10^3^/mm^3^	−2.99 ± 1.36	−5.69, −0.28	−0.20	0.031	3	0.195
t-Gestational age, wk	0.17 ± 0.07	0.02, 0.31	0.21	0.026	4	0.185
t-Wood smoke, h/d	−0.20 ± 0.11	−0.43, 0.02	−0.16	0.078	5	0.107
Constant	0.04 ± 0.13	−0.22, 0.31		0.733	Overall fit statistic = 0.277	

* Winsorized variable: 1 extremely high observation of plasma volume was replaced. ^1^ HCAZ model (*p* = 0.0021, adj. R^2^ = 0.15, *n* = 97, VIF = 1.12, condition number= 1.64. Variables with ≥500 bootstrap repetitions excluded by MFP process: BMI. Transformation of covariates (t): Gestational age (GA) = GA − 39.48453608; Weeks post-partum = weeks post-partum − 11.57731959; Parity (*n*) = parity − 3.536082474; plasma volume = plasma volume − 19.91539201; plateletcrit = plateletcrit − 28.35463923; ferritin = ferritin − 30.68969062. ^2^ LAZ model (*p* < 0.0001, adj. R^2^ = 0.33, *n* = 95, VIF = 1.06, condition number = 1.38). Variables with ≥ 500 bootstrap repetitions excluded by MFP process: BMI, IL-17, IGF-1. Transformation of covariates (t): gestational age (GA) = GA − 39.50526316; plasma volume = plasma volume − 19.90834408; months on iron supplements = months taking iron − 5.084210526; multiple nutrient supplement (MNS) = MNS − 0.9578947368; platelets= platelets − 0.3244947364; vitamin D = vitamin D − 43.08842097. ^3^ WAZ model (*p* < 0.0001, adj. R^2^ = 0.24, *n* = 96, VIF = 1.03, condition number = 1.23, Cook’s distance < 1.00). Variables with ≥ 500 bootstrap excluded by MFP process: BMI, hemoglobin, IL6, winsorized IL-17, MCP-1, vitamin D, IGF-1. Transformation of covariates (t): gestational age (GA) = GA − 39.47916667; wood smoke = wood smoke − 2.239583333; plasma volume = plasma volume − 19.89900244; animal-source foods = animal-source foods − 3.125; platelets = platelets – 0.3246249997.

## Data Availability

Data available on request due to privacy and ethical restrictions. The data presented in this study are available on request from the corresponding author. The data are not publicly available because participants did not sign an informed consent stating that data would be publicly available; neither was this possibility discussed with the Indigenous communities or the Ethical Board in Panama.

## Supplementary Materials

The following supporting information can be downloaded at: https://www.mdpi.com/article/10.3390/nu14173497/s1, Table S1: Comparison of maternal blood, inflammation, iron, and other nutritional indicators by protein status as measured by retinol binding protein (RBP); Table S2: MFP linear regression model for log-platelet counts. Table S3: Spearman correlation matrix for weeks post-partum and (A) iron status indicators, (B) serum nutritional indicators, (C) inflammation biomarkers.

## References

[1] Milman N. (2011). Postpartum anemia I: Definition, prevalence, causes, and consequences. Ann. Hematol..

[2] Abe S.K., Balogun O.O., Ota E., Takahashi K., Mori R. (2016). Supplementation with multiple micronutrients for breastfeeding women for improving outcomes for the mother and baby. Cochrane Database Syst. Rev..

[3] Green R. (2017). Vitamin B12 deficiency from the perspective of a practicing hematologist. Blood.

[4] Michelazzo F.B., Oliveira J.M., Stefanello J., Luzia L.A., Rondo P.H. (2013). The influence of vitamin A supplementation on iron status. Nutrients.

[5] Azizi-Soleiman F., Vafa M., Abiri B., Safavi M. (2016). Effects of iron on vitamin D metabolism: A systematic review. Int. J. Prev. Med..

[6] Bernát I., Bernát I. (1983). Protein-Deficiency Anemia. Iron Metabolism.

[7] Fondu P., Hariga-Muller C., Mozes N., Neve J., Van Steirteghem A., Mandelbaum I.M. (1978). Protein-energy malnutrition and anemia in Kivu. Am. J. Clin. Nutr..

[8] Wirth J.P., Woodruff B.A., Engle-Stone R., Namaste S.M., Temple V.J., Petry N., Macdonald B., Suchdev P.S., Rohner F., Aaron G.J. (2017). Predictors of anemia in women of reproductive age: Biomarkers Reflecting Inflammation and Nutritional Determinants of Anemia (BRINDA) project. Am. J. Clin. Nutr..

[9] Warrier R.P., Dole M.G., Warrier J., Suskind R.M., Suskind R.M., Lewinter-Suskind L. (1990). The Anemia of Malnutrition. The Malnourished Child.

[10] Guralnik J.M., Eisenstaedt R.S., Ferrucci L., Klein H.G., Woodman R.C. (2004). Prevalence of anemia in persons 65 years and older in the United States: Evidence for a high rate of unexplained anemia. Blood.

[11] Nemeth E., Ganz T. (2014). Anemia of inflammation. Hematol. Oncol. Clin. N. Am..

[12] Pasricha S.R., Atkinson S.H., Armitage A.E., Khandwala S., Veenemans J., Cox S.E., Eddowes L.A., Hayes T., Doherty C.P., Demir A.Y. (2014). Expression of the iron hormone hepcidin distinguishes different types of anemia in African children. Sci. Transl. Med..

[13] Bah A., Muhammad A.K., Wegmuller R., Verhoef H., Goheen M.M., Sanyang S., Danso E., Sise E.A., Pasricha S.R., Armitage A.E. (2019). Hepcidin-guided screen-and-treat interventions against iron-deficiency anaemia in pregnancy: A randomised controlled trial in The Gambia. Lancet Glob. Health.

[14] Gebreegziabher T., Roice T., Stoecker B.J. (2020). Chronic inflammation was a major predictor and determinant factor of anemia in lactating women in Sidama zone southern Ethiopia: A cross-sectional study. PLoS ONE.

[15] WHO, CDC Assessing the Iron Status of Populations. Apps.who.int/iris/bitstream/10665/75368/1/9789241596107_eng.pdf?ua=1&ua=1.

[16] Dignass A., Farrag K., Stein J. (2018). Limitations of Serum Ferritin in Diagnosing Iron Deficiency in Inflammatory Conditions. Int. J. Chronic Dis..

[17] WHO C-Reactive Protein Concentrations as a Marker of Inflammation or Infection for Interpreting Biomarkers of Micronutrient Status. http://apps.who.int/iris/bitstream/10665/133708/1/WHO_NMH_NHD_EPG_14.7_eng.pdf?ua=1.

[18] Namaste S.M., Aaron G.J., Varadhan R., Peerson J.M., Suchdev P.S. (2017). Methodologic approach for the Biomarkers Reflecting Inflammation and Nutritional Determinants of Anemia (BRINDA) project. Am. J. Clin. Nutr..

[19] Zhao A., Cao S., Gao H., Xiao Q., Win N., Zhang Y. (2016). Anemia among lactating mothers in Kokang, Myanmar. Southeast Asian J. Trop. Med. Public Health.

[20] Nairz M., Theurl I., Wolf D., Weiss G. (2016). Iron deficiency or anemia of inflammation? Differential diagnosis and mechanisms of anemia of inflammation. Wien Med. Wochenschr..

[21] Beguin Y. (2003). Soluble transferrin receptor for the evaluation of erythropoiesis and iron status. Clin. Chim. Acta.

[22] Rohner F., Namaste S.M., Larson L.M., Addo O.Y., Mei Z., Suchdev P.S., Williams A.M., Sakr Ashour F.A., Rawat R., Raiten D.J. (2017). Adjusting soluble transferrin receptor concentrations for inflammation: Biomarkers Reflecting Inflammation and Nutritional Determinants of Anemia (BRINDA) project. Am. J. Clin. Nutr..

[23] Mujica-Coopman M.F., Brito A., Lopez de Romana D., Rios-Castillo I., Coris H., Olivares M. (2015). Prevalence of Anemia in Latin America and the Caribbean. Food Nutr. Bull..

[24] Gonzalez-Fernandez D., Koski K.G., Sinisterra O.T., Del Carmen Pons E., Murillo E., Scott M.E. (2015). Interactions among urogenital, intestinal, skin, and oral infections in pregnant and lactating panamanian Ngäbe women: A neglected public health challenge. Am. J. Trop. Med. Hyg..

[25] Gonzalez-Fernandez D., Pons E.D.C., Rueda D., Sinisterra O.T., Murillo E., Scott M.E., Koski K.G. (2017). C-reactive protein is differentially modulated by co-existing infections, vitamin deficiencies and maternal factors in pregnant and lactating indigenous Panamanian women. Infect. Dis. Poverty.

[26] Ministerio de Salud de Panamá Plan Nacional “Prevención y Control de las Deficiencias de Micronutrientes” 2008–2015. http://es.wfp.org/sites/default/files/es/file/plan__nacional_prevencion_y_control_de_las_deficiencias_de_micronutrientes_2008_2015.pdf.

[27] De León J., Barba A., Sinisterra O.T., Atencio A. II Nutritional Monitoring in MINSA Facilities, MONINUT 2017. https://www.google.com/url?sa=t&rct=j&q=&esrc=s&source=web&cd=&ved=2ahUKEwjvjdzKpcLvAhXaEVkFHTjmDYQQFjABegQIAxAD&url=https%3A%2F%2Fnutricionistaspanama.com%2Fwp-content%2Fuploads%2Fpublicaciones%2FINFORME_MNINUT.pdf&usg=AOvVaw1RMKpMtoQXF1SdTy2wqs8f.

[28] Panamanian Ministry of Health (2019). Sistema de Información Geográfico Interactivo de la Encuesta Nacional de Salud de Panamá (ENSPA). http://www.gorgas.gob.pa/SIGENSPA/Grafica_deplecion_ferropriva_anemia_MEF.html.

[29] Lewies A., Zandberg L., Baumgartner J. (2019). Interventions to prevent iron deficiency during the first 1000 days in low-income and middle-income countries: Recent advances and challenges. Curr. Opin. Clin. Nutr. Metab. Care.

[30] Dessie Z.B., Fentie M., Abebe Z., Ayele T.A., Muchie K.F. (2019). Maternal characteristics and nutritional status among 6-59 months of children in Ethiopia: Further analysis of demographic and health survey. BMC Pediatr..

[31] Li C., Solomons N.W., Scott M.E., Koski K.G. (2016). Minerals and Trace Elements in Human Breast Milk Are Associated with Guatemalan Infant Anthropometric Outcomes within the First 6 Months. J. Nutr..

[32] Saso A., Blyuss O., Munblit D., Faal A., Moore S.E., Le Doare K. (2018). Breast Milk Cytokines and Early Growth in Gambian Infants. Front. Pediatr..

[33] Tuaillon E., Viljoen J., Dujols P., Cambonie G., Rubbo P.A., Nagot N., Bland R.M., Badiou S., Newell M.L., Van de Perre P. (2017). Subclinical mastitis occurs frequently in association with dramatic changes in inflammatory/anti-inflammatory breast milk components. Pediatric Res..

[34] Wren-Atilola H.M., Solomons N.W., Scott M.E., Koski K.G. (2021). Infant Anthropometry and Growth Velocity Before 6 Months are Associated with Breastfeeding Practices and the Presence of Subclinical Mastitis and Maternal Intestinal Protozoa in Indigenous Communities in Guatemala. Curr. Dev. Nutr..

[35] Brannon P.M., Taylor C.L. (2017). Iron Supplementation during Pregnancy and Infancy: Uncertainties and Implications for Research and Policy. Nutrients.

[36] Halpenny C.M., Koski K.G., Valdes V.E., Scott M.E. (2012). Prediction of child health by household density and asset-based indices in impoverished indigenous villages in rural Panama. Am. J. Trop. Med. Hyg..

[37] Keller U. (2019). Nutritional Laboratory Markers in Malnutrition. J. Clin. Med..

[38] World Health Organization WHO Guideline on Use of Ferritin Concentrations to Assess Iron Status in Individuals and Populations. https://apps.who.int/iris/handle/10665/331505.

[39] Abbassi-Ghanavati M., Greer L.G., Cunningham F.G. (2009). Pregnancy and laboratory studies: A reference table for clinicians. Obstet. Gynecol..

[40] World Health Organization Iron Deficiency Anaemia: Assessment, Prevention and Control: A Guide for Programme Managers. https://www.who.int/nutrition/publications/micronutrients/anaemia_iron_deficiency/WHO_NHD_01.3/en/.

[41] De Benoist B. (2008). Conclusions of a WHO Technical consultation on folate and vitamin B12 deficiencies. Food Nutr. Bull..

[42] Balvers M.G., Brouwer-Brolsma E.M., Endenburg S., de Groot L.C., Kok F.J., Gunnewiek J.K. (2015). Recommended intakes of vitamin D to optimize health, associated circulating 25-hydroxyvitamin D concentrations, and dosing regimens to treat deficiency: Workshop report and overview of current literature. J. Nutr. Sci..

[43] Wiseman E.M., Bar-El Dadon S., Reifen R. (2016). The vicious cycle of vitamin A deficiency: A review. Crit. Rev. Food Sci. Nutr..

[44] Sinisterra O.T., Pons E.d.C., Fontes F., Lagrutta F., Carrasco Y., Olivares M. (2012). Evaluación del programa de suplementación con hierro en Panamá. Avances de Investigación en Seguridad Alimentaria y Nutricional (SAN).

[45] Jansson L., Nilsson B. (1983). Serum retinol and retinol-binding protein in mothers and infants at delivery. Biol. Neonate.

[46] Fujita M., Brindle E., Rocha A., Shell-Duncan B., Ndemwa P., O’Connor K.A. (2009). Assessment of the relative dose-response test based on serum retinol-binding protein instead of serum retinol in determining low hepatic vitamin A stores. Am. J. Clin. Nutr..

[47] Gamble M.V., Ramakrishnan R., Palafox N.A., Briand K., Berglund L., Blaner W.S. (2001). Retinol binding protein as a surrogate measure for serum retinol: Studies in vitamin A-deficient children from the Republic of the Marshall Islands. Am. J. Clin. Nutr..

[48] Feranchak A.P., Gralla J., King R., Ramirez R.O., Corkill M., Narkewicz M.R., Sokol R.J. (2005). Comparison of indices of vitamin A status in children with chronic liver disease. Hepatology.

[49] World Health Organization Report: Priorities in the Assessment of Vitamin A and Iron Status in Populations, Panama City, Panama, 15–17 September 2010. https://apps.who.int/iris/handle/10665/75334.

[50] Panamanian Ministry of Health Normas técnicas y administrativas del programa de salud integral del niño y la niña desde el nacimiento a los 9 años de edad. https://siteal.iiep.unesco.org/bdnp/2447/normas-tecnicas-administrativas-programa-salud-integral-nino-nina-desde-nacimiento-9-anos.

[51] World Health Organization A Healthy Lifestyle-WHO Recommendations. https://www.who.int/europe/news-room/fact-sheets/item/a-healthy-lifestyle---who-recommendations.

[52] Stieglitz E., Huang J. Plasmapheresis Technique. https://emedicine.medscape.com/article/1895577-technique.

[53] Hauser R.G., Kwon R.J., Ryder A., Cheng C., Charifa A., Tormey C. (2020). Transfusion medicine equations made internet accessible. Transfus. Med. Rev..

[54] Bruinse H.W., van der Berg H., Haspels A.A. (1985). Maternal serum folacin levels during and after normal pregnancy. Eur. J. Obstet. Gynecol. Reprod. Biol..

[55] Vidmar S.I., Cole T.J., Pan H. (2013). Standardizing anthropometric measures in children and adolescents with functions for egen: Update. Stata J..

[56] Mannan H. (2017). A practical Application of a simple bootstrapping method for assessing predictors selected for epidemiologic risk models using automated variable selection. Int. J. Stat. Appl..

[57] Royston P., Sauerbrei W. (2016). mfpa: Extension of mfp using the ACD covariate transformation for enhanced parametric multivariable modeling. Stata J..

[58] Royston P., Sauerbrei W. (2008). Multivariable Model-Building: A Pragmatic Approach to Regression Analysis based on Fractional Polynomials for Modelling Continuous Variables.

[59] Hosmer D.W., Lemeshow S., Sturdivant R.X. (2013). Model-Building Strategies and Methods for Logistic Regression. Applied Logistic Regression.

[60] Green S.B. (1991). How many subjects does it take to do a regression analysis. Multivariate Behav. Res..

[61] Ludwig-Mayerhofer W. Winsorizing and Trimming. https://wlm.userweb.mwn.de/Stata/wstatwin.htm.

[62] Grömping U. (2007). Estimators of relative importance in linear regression based on variance decomposition. Am. Stat..

[63] Nehring S.M., Goyal A., Bansal P., Patel B.C. (2021). C Reactive Protein. StatPearls.

[64] Ravin K.A., Loy M. (2016). The eosinophil in infection. Clin. Rev. Allergy Immunol..

[65] Bonaccio M., Di Castelnuovo A., Pounis G., De Curtis A., Costanzo S., Persichillo M., Cerletti C., Donati M.B., de Gaetano G., Iacoviello L. (2016). A score of low-grade inflammation and risk of mortality: Prospective findings from the Moli-sani study. Haematologica.

[66] Mishra S., Jaiswar S., Saad S., Tripathi S., Singh N., Deo S., Agarwal M., Mishra N. (2021). Platelet indices as a predictive marker in neonatal sepsis and respiratory distress in preterm prelabor rupture of membranes. Int. J. Hematol..

[67] Namaste S.M., Rohner F., Huang J., Bhushan N.L., Flores-Ayala R., Kupka R., Mei Z., Rawat R., Williams A.M., Raiten D.J. (2017). Adjusting ferritin concentrations for inflammation: Biomarkers Reflecting Inflammation and Nutritional Determinants of Anemia (BRINDA) project. Am. J. Clin. Nutr..

[68] Ganz T. (2019). Anemia of inflammation. N. Engl. J. Med..

[69] Schumann K., Solomons N.W. (2017). Perspective: What makes it so difficult to mitigate worldwide anemia prevalence?. Adv. Nutr..

[70] Weiss G., Ganz T., Goodnough L.T. (2019). Anemia of inflammation. Blood.

[71] Jorgensen J.M., Yang Z., Lönnerdal B., Chantry C.J., Dewey K.G. (2017). Plasma ferritin and hepcidin are lower at 4 months postpartum among women with elevated C-reactive protein or α1-acid glycoprotein. J. Nutr..

[72] Vecchi C., Montosi G., Garuti C., Corradini E., Sabelli M., Canali S., Pietrangelo A. (2014). Gluconeogenic signals regulate iron homeostasis via hepcidin in mice. Gastroenterology.

[73] Troutt J.S., Rudling M., Persson L., Ståhle L., Angelin B., Butterfield A.M., Schade A.E., Cao G., Konrad R.J. (2012). Circulating human hepcidin-25 concentrations display a diurnal rhythm, increase with prolonged fasting, and are reduced by growth hormone administration. Clin. Chem..

[74] Skikne B.S. (2008). Serum transferrin receptor. Am. J. Hematol..

[75] Canete A., Cano E., Munoz-Chapuli R., Carmona R. (2017). Role of vitamin A/retinoic acid in regulation of embryonic and adult hematopoiesis. Nutrients.

[76] Santos E.W., Oliveira D.C., Silva G.B., Tsujita M., Beltran J.O., Hastreiter A., Fock R.A., Borelli P. (2017). Hematological alterations in protein malnutrition. Nutr. Rev..

[77] Tanumihardjo S.A., Russell R.M., Stephensen C.B., Gannon B.M., Craft N.E., Haskell M.J., Lietz G., Schulze K., Raiten D.J. (2016). Biomarkers of Nutrition for Development (BOND)-Vitamin A Review. J. Nutr..

[78] Haidar J., Muroki N.M., Omwega A.M., Ayana G. (2003). Malnutrition and iron deficiency in lactating women in urban slum communities from Addis Ababa, Ethiopia. East Afr. Med. J..

[79] Vinoy S., Rosetta L., Mascie-Taylor C.G. (2000). Repeated measurements of energy intake, energy expenditure and energy balance in lactating Bangladeshi mothers. Eur. J. Clin. Nutr..

[80] Roba K.T., O’Connor T.P., Belachew T., O’Brien N.M. (2015). Seasonal variation in nutritional status and anemia among lactating mothers in two agro-ecological zones of rural Ethiopia: A longitudinal study. Nutrition.

[81] Livingstone C. (2013). Insulin-like growth factor-I (IGF-I) and clinical nutrition. Clin. Sci..

[82] Succurro E., Arturi F., Caruso V., Rudi S., Sciacqua A., Andreozzi F., Hribal M.L., Perticone F., Sesti G. (2011). Low insulin-like growth factor-1 levels are associated with anaemia in adult non-diabetic subjects. Thromb. Haemost..

[83] Brent B., Obonyo N., Akech S., Shebbe M., Mpoya A., Mturi N., Berkley J.A., Tulloh R.M.R., Maitland K. (2019). Assessment of myocardial function in Kenyan children with severe, acute malnutrition: The Cardiac Physiology in Malnutrition (CAPMAL) study. JAMA Netw. Open.

[84] Caregaro L., Di Pascoli L., Favaro A., Nardi M., Santonastaso P. (2005). Sodium depletion and hemoconcentration: Overlooked complications in patients with anorexia nervosa?. Nutrition.

[85] Ali-Baya G., Zenile E., Aikins B.O., Amoaning R.E., Simpong D.L., Adu P. (2021). Poor haemoglobin-haematocrit agreement in apparently healthy adult population; a cross-sectional study in Cape Coast Metropolis, Ghana. Heliyon.

[86] Bardosono S., Morin C., Guelinckx I., Pohan R. (2017). Pregnant and breastfeeding women: Drinking for two?. Ann. Nutr. Metab..

[87] Heen E., Yassin A.A., Madar A.A., Romøren M. (2021). Estimates of fluid intake, urine output and hydration-levels in women from Somaliland: A cross-sectional study. J. Nutr. Sci..

[88] World Health Organization Guideline: Iron Supplementation in Postpartum Women. https://www.who.int/nutrition/publications/micronutrients/guidelines/daily_iron_supp_postpartum_women/en/.

[89] Gautam C.S., Saha L., Sekhri K., Saha P.K. (2008). Iron deficiency in pregnancy and the rationality of iron supplements prescribed during pregnancy. Medscape J. Med..

[90] Berglund S.K., Domellöf M. (2021). Iron deficiency in infancy: Current insights. Curr. Opin. Clin. Nutr. Metab. Care.

[91] Pérez-Escamilla R., Buccini G.S., Segura-Pérez S., Piwoz E. (2019). Perspective: Should exclusive breastfeeding still be recommended for 6 months?. Adv. Nutr..

[92] Taylor C.L., Brannon P.M. (2017). Introduction to workshop on iron screening and supplementation in iron-replete pregnant women and young children. Am. J. Clin. Nutr..

[93] Nairz M., Weiss G. (2019). Infections associated with iron administration. Met. Ions Life Sci..

[94] Wahed M.A., Alvarez J.O., Khaled M.A., Mahalanabis D., Rahman M.M., Habte D. (1995). Comparison of the modified relative dose response (MRDR) and the relative dose response (RDR) in the assessment of vitamin A status in malnourished children. Am. J. Clin. Nutr..

[95] González-Fernández D., Nemeth E., Pons E.d.C., Rueda D., Sinisterra O.T., Murillo E., Sangkhae V., Starr L.M., Scott M.E., Koski K.G. (2021). INTERGROWTH-21 identifies high prevalence of low symphysis-fundal height in indigenous pregnant women experiencing multiple infections, nutrient deficiencies and inflammation: The MINDI cohort. Curr. Dev. Nutr..

[96] Asare H., Rosi A., Faber M., Smuts C.M., Ricci C. (2022). Animal-source foods as a suitable complementary food for improved physical growth in 6 to 24-month-old children in low- and middle-income countries: A systematic review and meta-analysis of randomised controlled trials. Br. J. Nutr..

[97] De Caballero E.A.E. (2003). Evaluation on the Acceptability and Consumption of Nutricrema in the Republic of Panama. Rev. Chil. Nutr..

[98] Panamanian Ministry of Health Prácticas de cuidado y alimentación infantil en las Comarcas indígenas de Kuna-Yala, Ngöbe-Buglé, Emberá-Wounaan y los distritos de Cañazas y Las Palmas (Provincia de Veraguas). https://www.google.com/url?sa=t&rct=j&q=&esrc=s&source=web&cd=&ved=2ahUKEwjY2qCokP_wAhXFc98KHYSSA1kQFjAHegQIDBAE&url=https%3A%2F%2Fwww.paho.org%2Fhq%2Findex.php%3Foption%3Dcom_docman%26view%3Ddownload%26category_slug%3Dexperiencias-ver-mas-sitio-web-ingles-4986%26alias%3D19856-2009-panama-856%26Itemid%3D270&usg=AOvVaw2_coNrI3xk0kgLJSK9ns5u.

[99] Brabin L., Brabin B.J., Gies S. (2013). Influence of iron status on risk of maternal or neonatal infection and on neonatal mortality with an emphasis on developing countries. Nutr. Rev..

[100] Sinisterra O.F.F., Lagrutta F., Olivares M. Situación de deficiencia de hierro y anemia. https://www.unicef.org/panama/spanish/Hierro.pdf.

[101] Wagner C.L., Hollis B.W. (2020). Early-life effects of vitamin D: A focus on pregnancy and lactation. Ann. Nutr. Metab..

[102] Aghajafari F., Nagulesapillai T., Ronksley P.E., Tough S.C., O’Beirne M., Rabi D.M. (2013). Association between maternal serum 25-hydroxyvitamin D level and pregnancy and neonatal outcomes: Systematic review and meta-analysis of observational studies. BMJ.

[103] Roth D.E., Morris S.K., Zlotkin S., Gernand A.D., Ahmed T., Shanta S.S., Papp E., Korsiak J., Shi J., Islam M.M. (2018). Vitamin D supplementation in pregnancy and lactation and infant growth. NEJM.

[104] Dawodu A., Salameh K.M., Al-Janahi N.S., Bener A., Elkum N. (2019). The effect of high-dose postpartum maternal vitamin D supplementation alone compared with maternal plus infant vitamin D supplementation in breastfeeding infants in a high-risk population a randomized controlled trial. Nutrients.

[105] Grant W.B., Al Anouti F., Moukayed M. (2020). Targeted 25-hydroxyvitamin D concentration measurements and vitamin D supplementation can have important patient and public health benefits. Eur. J. Clin. Nutr..

