# Supplementary material for: Multiple Indicators of Undernutrition, Infection, and Inflammation in Lactating Women Are Associated with Maternal Iron Status and Infant Anthropometry in Panama: The MINDI Cohort
Source: Nutrients. 2022 Aug 25;14(17):3497. doi: 10.3390/nu14173497 (PMC9460351; doi:10.3390/nu14173497)
Supplement: Supplementary file 1 [file nutrients-14-03497-s001.zip › nutrients-1801564-supplementary (1)-done.pdf]

nutrients

MDPI

Multiple Indicators of Undernutrition, Infection and Inflammation in Lactating Women Are Associated with Maternal Iron Status and Infant Anthropometry in Panama: The MINDI Cohort

Doris González-Fernández, Elizabeta Nemeth, Emérita del Carmen Pons, Odalis Teresa Sinisterra, Delfina Rueda, Lisa Starr, Veena Sangkhae, Enrique Murillo, Marilyn E. Scott and Kristine G. Koski

Supplementary Material

Table S1. Comparison of maternal blood, inflammation, iron and other nutritional indicators by protein status as measured by retinol binding protein (RBP): protein deficient (RBP<30 mg/L) and protein sufficient (RBP≥30 mg/L). Means ± SD are presented for normally-distributed variables, and medians with interquartile regions for non-normally distributed variables.

	**RBP <30 mg/L n=36**	**RBP ≥30 mg/L n=63**	**p**
**RBC indices**			
Hemoglobin (g/L)	114.8 ± 1.7	119.1 ± 1.4	**0.031**
Hematocrit (%)*	36.9 ± 0.5	38.1 ± 0.4	**0.023**
Total RBC x10^6/mm^3	3.98 ± 0.07	4.10 ± 0.04	0.07
MCV, fL	92.9 ± 0.8	92.7 ± 0.6	0.40
MCH, pg	28.9 ± 0.3	29.1 ± 0.2	0.34
MCHC, g/L	311.3 ± 2.0	314.2 ± 1.3	0.11
RDW-CV, %	14.0 ± 0.2	13.7 ± 0.1	0.14
**WBC indices** x10^3/mm^3			
Total WBC	8.9 ± 3.4	7.8 ± 2.1	**0.004**
Neutrophils	4.8 (3.7 – 6.7)	4.0 (3.4 – 5.0)	0.039
Lymphocytes	2.5 ± 1.0	2.4 ± 8.1	0.21
Monocytes	0.40 (0.33 – 0.46)	0.34 (0.31 – 0.40)	0.016
Eosinophils	0.63 (0.36 – 0.91)	0.59 (0.33 – 0.91)	0.70
Basophils	0.05 (0.03 – 0.06)	0.04 (0.03 – 0.06)	0.58
**Iron indicators**			
Ferritin, μg/L	23.0 (13.6 – 34.2)	24.2 (13.9 – 37.6)	0.68
sTfR, mg/L	8.59 (6.62 – 10.76)	7.51 (5.87 – 9.37)	0.14
Serum iron, μmol/L	9.88 (6.77 – 12.0)	10.44 (7.45 – 14.34)	0.31
Hepcidin, μg/L	15.4 (12.0 – 21.1)	15.7 (11.3 – 20.6)	0.96
**Inflammation indicators**			
CRP, mg/L	1.30 (0.50 – 2.75)	1.40 (0.50 – 4.10)	0.66
NLR	1.90 (1.55 – 2.48)	1.75 (1.32 – 2.31)	0.29
**Cytokines (pg/mL)**			
IL-1β	1.01 (0.02 – 2.04)	0.02 (0.02 – 0.99)	0.17
IL-4	2.44 (0.24 – 3.80)	2.06 (0.24 – 5.25)	0.70
IL-6, median (min-max)	1.60 (1.60 – 88.9)	1.60 (1.60 – 51.90)	0.67
IL-10	0.32 (0.13 – 2.00)	0.32 (0.07 – 1.11)	0.23
IL-12	1.57 (0.37 – 5.49)	0.88 (0.06 – 6.11)	0.56
IL-13	0.78 (0.28 – 0.93)	0.93 (0.93 – 1.60)	**0.0001**
IL-17	0.79 (0.56 – 1.81)	0.93 (0.47 – 1.70)	0.77
TNF-α	5.17 (0.50 – 11.02)	4.38 (2.69 – 7.74)	0.70
IFN-γ	1.80 (0.69 – 3.72)	1.41 (0.05 – 3.48)	0.29
MCP-1	322.3 (285.6 – 429.1)	314.2 (238.0 – 388.2)	0.61
**Nutritional indicators**			

Retinol/RBP ratio	1.27 (1.06 – 2.14)	0.59 (0.37 – 0.86)	**0.0001**
Folic acid, nmol/L	11.2 (8.3 – 14.1)	12.4 (10.1 – 16.5)	0.07
Vitamin B_{12}, pmol/L	151.5 (121.0 – 195.5)	153.0 (129.0 – 187.0)	0.99
Vitamin A, μmol/L	1.39 (1.20 – 1.56)	1.41 (1.12 – 1.88)	0.45
IGF-1, ng/mL	28.8 (25.6 – 34.3)	31.8 (27.6 – 34.6)	0.34

*One influential observation was removed. P values<0.05 are in bold.

Abbreviations: CRP, C-reactive protein; IGF-1, insulin-like growth factor; IL, interleukin; MCH, mean corpuscular hemoglobin; MCHC, mean corpuscular hemoglobin concentration; MCP-1, monocyte chemoattractant protein-1; MCV, mean corpuscular volume; NLR, neutrophil-lymphocyte ratio; RBC, red blood cells; RBP, retinol-binding protein; RDW-CV: red cell deviation width - coefficient of variation; SD, standard deviation; sTfR, serum transferrin receptor; WBC, white blood cells.

Table S2. MFP linear regression model for log-platelet counts.

Log-platelets [1]	Coef ± SE	95% CI	β	p-value	Ranking	Standardized dominance
t- CRP, mg/L	0.02 ± 0.007	0.01, 0.04	0.30	0.001	1	0.283
t- Weeks post-partum	-0.01 ± 0.003	-0.02, -0.005	-0.29	<0.0001	2	0.234
t- Cups of coffee/d	0.07 ± 0.02	0.03, 0.11	0.29	<0.0001	3	0.165
t- Serum iron, μmol/L	-0.01 ± 0.005	-0.02, -0.0002	-0.17	0.044	4	0.103
t- Scabies, presence	0.17 ± 0.08	0.01, 0.33	0.17	0.037	5	0.078
t- Bacteriuria, presence	-0.18 ± 0.07	-0.32, -0.04	-0.20	0.011	6	0.077
t- Urinary specific gravity	0.01 ± 0.003	0.002, 0.02	0.21	0.008	7	0.060
Constant	5.74 ± 0.02	5.69, 5.79		<0.0001	Overall fit statistic=0.499	

[1] Log platelets model (n=94, p< 0.0001, adj. R^2= 0.46, VIF=1.11, condition number=1.83). Variables with ≥500 bootstrap repetitions excluded by MFP process: fieldwork, exclusive breastfeeding, hematocrit, monocytes, hemoglobin, IL-17 square root, vitamin B_{12}.

Transformation of covariates:.

t- Weeks post-partum = weeks post-partum – 11.67021277.

t- Cups of coffee/d = cups of coffee/d – 2.138297872.

t- C-reactive protein (CRP) = CRP – 2.97765958.

t- Urinary specific gravity = urinary specific gravity – 1017.87234.

t- Serum iron = serum iron – 10.69734034.

Table S3. Spearman correlation matrix for weeks post-partum and (A) iron status indicators, (B) serum nutritional indicators, (C) inflammation biomarkers.

**A.**		**Weeks post-partum**	**Hemoglobin, g/L**	**Hematocrit, %**	**Ferritin, μg/L**	**Serum iron, μmol/L**	**sTfR, mg/L**	**Hepcidin, μg/L**
**Weeks post-partum**		1						
**Hemoglobin, g/L**	r	0.2825	1					
	p	0.0046						
**Hematocrit, %**	r	0.281	0.9299	1				
	p	0.0048	0					
**Ferritin, μg/L**	r	-0.0921	0.3143	0.2721	1			
	p	0.3645	0.0015	0.0064				
**Serum iron, μmol/L**	r	0.0665	0.4352	0.3897	0.3968	1		
	p	0.5134	0	0.0001	0			

**sTfR, mg/L**	r	-0.0071	-0.0474	0.0287	-0.2864	-0.2301	1	
	p	0.9446	0.641	0.7783	0.0041	0.0219		
**Hepcidin, µg/L**	r	-0.1185	0.2733	0.2571	0.6436	0.3178	-0.1552	1
	p	0.2425	0.0062	0.0102	0	0.0013	0.1252	

**B.**		**Weeks post-partum**	**Folic acid, nmol/L**	**Vitamin B-12, pmol/L**	**Vitamin A, µmol/L**	**RBP, mg/L**	**IGF-1, pmol/L**
**Weeks post-partum**		1					
**Folic acid, nmol/L**	r	0.3276	1				
	p	0.0013					
**Vitamin B-12, pmol/L**	r	-0.159	-0.077	1			
	p	0.1258	0.4608				
**Vitamin A, µmol/L**	r	-0.0141	0.2144	-0.1096	1		
	p	0.8928	0.038	0.293			
**RBP, mg/L**	r	0.1067	0.1519	0.0051	0.0523	1	
	p	0.3061	0.1439	0.9612	0.6167		
**IGF-1, pmol/L**	r	0.0103	0.0067	0.0829	0.0626	-0.0483	1
	p	0.9215	0.9488	0.427	0.5488	0.6439	

		**Weeks PP**	**CRP**	**IL-1β**	**IL4**	**IL6**	**IL10**	**IL12**	**IL13**	**IL17**	**IFN-γ**	**TNF-α**	**MCP-1**
**Weeks PP**		1											
**CRP**	r	-0.275	1										
	p	0.006											
**IL-1β**	r	-0.086	0.023	1									
	p	0.394	0.817										
**IL-4**	r	-0.203	0.205	0.229	1								
	p	0.044	0.042	0.023									
**IL-6**	r	0.115	0.032	0.158	0.180	1							
	p	0.257	0.749	0.118	0.075								
**IL-10**	r	-0.214	-0.033	0.333	0.185	0.095	1						
	p	0.033	0.742	0.001	0.067	0.352							
**IL-12**	r	-0.197	0.016	0.397	0.283	-0.032	0.329	1					
	p	0.050	0.876	0	0.004	0.753	0.001						
**IL-13**	r	0.275	-0.017	-0.347	-0.046	0.084	-0.453	-0.237	1				
	p	0.006	0.870	0.0004	0.653	0.410	0	0.018					
**IL-17**	r	-0.110	0.061	0.242	0.391	0.236	0.252	0.231	-0.006	1			
	p	0.279	0.551	0.016	0.0001	0.019	0.012	0.022	0.949				
**INF-γ**	r	-0.182	0.049	0.490	0.324	0.148	0.271	0.404	-0.114	0.608	1		
	p	0.072	0.629	0	0.001	0.144	0.007	0	0.261	0			
**TNF-α**	r	0.037	0.069	0.184	0.305	0.387	0.164	0.155	-0.046	0.210	0.215	1	

	p	0.716	0.496	0.068	0.002	0.0001	0.104	0.125	0.647	0.037	0.033		
**MCP-1**	r	-0.230	-0.051	0.299	0.288	0.154	0.259	0.180	-0.158	0.180	0.262	0.253	1
	p	0.022	0.615	0.003	0.004	0.128	0.010	0.075	0.118	0.076	0.009	0.012	

PP: post-partum.
